# Physics-informed gaussian process regression for reproducible and uncertainty-aware CO_2_ injectivity prediction

**DOI:** 10.1371/journal.pone.0352178

**Published:** 2026-07-01

**Authors:** Shamsuddeen Adamu, Hitham Alhussian, Said Jadid Abdulkadir, Majdy Mohamed Eltayeb Eltahir, Sallam O. F. Khairy, Gasim Hayder, Mahdi Ali Lathbl, Hassan Salisu Mohammed, Abdulrazak Oladeji Adekunle, Gbolagade Kamaldeen, Samaila Musa Abdullahi, Yahaya Saidu

**Affiliations:** 1 Computer Information Science Department, Universiti Teknologi PETRONAS, Seri Iskandar, Malaysia; 2 IAIICT, Ahmadu Bello University, Zaria, Nigeria; 3 King Khalid University, Applied College of Muhayil Asir, Abha, Saudi Arabia; 4 Department of Information Systems, College of Economics, Management and Information Systems, University of Nizwa, Nizwa, Oman; 5 Environmental Engineering Department, University of Nizwa, Nizwa, Oman; 6 Department of Cyber Security, Faculty of Computing, Air Force Institute of Technology, AFIT, Kaduna, Nigeria; Henan Polytechnic University, CHINA

## Abstract

Reliable prediction of CO_2_ injectivity decline is essential for safe geological carbon storage, yet existing machine learning models often provide deterministic point predictions that lack uncertainty quantification. This paper presents a physics-informed Gaussian process regression (PC-GPR) framework for Relative Injectivity Change (RIC) prediction, embedding constraints derived from two independently grounded physical laws: the Derjaguin–Landau–Verwey–Overbeek (DLVO) colloidal monotonicity condition and the Civan–Kozeny–Carman permeability impairment model. Four GP variants are developed and benchmarked on a curated laboratory dataset (*n* = 44) under a three-tier validation protocol combining Leave-One-Out cross-validation, repeated *k*-fold cross-validation, and non-parametric bootstrap confidence intervals. Two complementary uncertainty quantification mechanisms are employed: GP posterior calibration via the Expected Calibration Error (ECE) and split-conformal prediction intervals. The GP-Base model achieves strong predictive performance (LOO *R*^2^ = 0.9401, 95% CI: [0.882, 0.978]) with well-calibrated uncertainty (ECE = 0.026) and reliable coverage (97.7% at the nominal 95% level). The PC-GPR-M variant reduces DLVO monotonicity violations to 1.5% across the input domain, demonstrating effective soft constraint enforcement. Operationally, the proposed framework translates predictive uncertainty into actionable injection scheduling guidance, identifying high-risk regions at salinity >30,000 ppm and jamming ratio >0.04. These results provide an uncertainty-aware baseline for future PIML research in subsurface carbon storage.

## 1. Introduction

Carbon capture and sequestration (CCS) has emerged as one of the most technically mature near-term pathways for large-scale mitigation of industrial CO_2_ emissions, with global injection capacity projected to exceed 1 Gt yr−1 by 2050 [1,2]. During CO_2_ injection into deep saline aquifers, fine-particle migration and progressive pore-throat plugging cause a reduction in formation injectivity that, left unmanaged, leads to wellbore pressure escalation, premature injection termination, and substantial economic loss [3]. The Relative Injectivity Change (RIC, %), defined as the fractional reduction in the injectivity index relative to its initial clean-brine value, is the primary field-deployable metric for quantifying this decline [4].

RIC is controlled by at least four interacting variables: brine salinity *S* (ppm), injection flow rate *Q* (ml min−1), jamming ratio *J* (–), and particle concentration $C_{p}$ (wt%), whose joint influence is nonlinear and governed by pore-scale electrostatic and hydrodynamic mechanisms [4]. Predictive models are therefore essential for injection schedule optimisation, well integrity monitoring, and regulatory compliance in active CCs operations.

### 1.1. Prior predictive models and their limitations

Data-driven approaches to RIC prediction have evolved through three generations. Response Surface Methodology (RSM) provided the first systematic relationship between inputs and RIC, attaining *R*^2^ = 0.985 on a single 80/20 partition of *n* = 44 laboratory measurements [5]. Support vector regression (SVR) improved accuracy to *R*^2^ = 0.992, and its genetic-algorithm-tuned variant (GA-SVR) represents the current state of the art with *R*^2^ = 0.9923, RMSE = 0.99, MAE = 0.50 on the same single partition [5].

Despite this impressive in-sample performance, three critical limitations characterise the existing literature and motivate the present work.

**L1 — No uncertainty quantification.** The published RIC model produces a single point prediction. No confidence interval, credible interval, or predictive distribution is reported for any individual forecast. For field-deployed CCS operations, a point prediction of RIC = 40% is operationally ambiguous without knowing whether the true value lies in [35%, 45%] (manageable) or [10%, 70%] (unacceptable risk).

**L2 — Single-split evaluation.** All published models are assessed on one fixed 80/20 partition of *n* = 44 observations. With only 9 held-out points, the standard error of *R*^2^ on the test set exceeds 0.05 units [6], making the reported figures statistically unreproducible. The authors’ preceding work [7] demonstrated empirically that repeated cross-validation and leave-one-out evaluation yield confidence intervals spanning ∼0.15 *R*^2^ units, confirming that single-split estimates are epistemically insufficient.

**L3 — No physical constraint enforcement.** All prior models are purely data-driven: they must rediscover the DLVO-governed salinity monotonicity and Kozeny–Carman pore-blocking kinetics from *n* = 44 points alone. Consequently, they may violate known physical ordering constraints in regions of sparse data, and their predictions carry no mechanistic interpretability beyond post-hoc feature importance analysis.

### 1.2. Physics-informed machine learning for geoscience

Physics-informed machine learning (PIML) addresses limitations L2 and L3 simultaneously by encoding known physical laws as inductive constraints, thereby reducing the effective hypothesis space and improving generalisation from small datasets [8]. Existing PIML applications to CO_2_ storage have predominantly focused at reservoir scale, where partial differential equations (PDES) govern pressure and saturation fields, with Fourier neural operator approaches showing particular promise [9–11]. The laboratory-scale fines-migration problem has a fundamentally different and more tractable constraint structure: ordinary differential equations (Civan) and monotonicity conditions (DLVO) rather than PDEs. No published work applies PIML to this problem at any scale.

Gaussian process regression (GPR) provides a natural framework for PIML on small datasets. As a kernel-based Bayesian non-parametric model, it provides native posterior predictive distributions without requiring distributional assumptions on residuals, and physical constraints can be embedded either through the prior mean function [12] or through virtual derivative observations [13]. Its $𝒪(n^{3})$ computational cost is manageable for *n* = 44.

Complementing GP posterior uncertainty, *conformal prediction* [14] provides distribution-free prediction intervals with guaranteed finite-sample marginal coverage, requiring no parametric assumptions on residual distributions. The combination of GP posterior and conformal intervals provides a two-layer uncertainty framework: the GP posterior reflects model uncertainty; conformal intervals provide coverage guarantees regardless of model misspecification.

### 1.3. Contributions

The primary goal of this work is to address three persistent gaps in CO_2_ injectivity prediction: (L1) absence of uncertainty quantification, (L2) reliance on single‑split evaluation, and (L3) lack of physical constraint enforcement. To close these gaps, the paper makes five contributions to geoscience machine learning and carbon storage research.

**C1. First PIML framework for fines‑migration injectivity.** Four Gaussian process variants embedding DLVO monotonicity and Civan permeability constraints are developed, systematically compared, and validated at the laboratory scale.
**C2. First calibrated uncertainty quantification for RIC prediction.** GP-Base achieves an Expected Calibration Error of 0.026 and, via split‑conformal prediction, attains 97.7% empirical coverage at the nominal 95% level — the first uncertainty‑quantified RIC predictor in the literature.
**C3. Soft DLVO monotonicity enforcement.** PC-GPR-M reduces violations of ∂RIC/∂S≥0 to 1.5% across 1,000 Latin Hypercube test points, providing the first physically regularised RIC model while explicitly reporting the residual violation rate.
**C4. Operational risk mapping.** The GP predictive surface is translated into an injection scheduling risk atlas that quantifies prediction interval widths at field‑relevant (*S*, *J*) combinations (Section 4.13), offering actionable guidance despite data sparsity.
**C5. Three‑tier validation protocol.** All eight models are evaluated under leave‑one‑out cross‑validation, repeated 5‑fold cross‑validation (*n*_rep_ = 20), and bootstrap confidence intervals (*B* = 2,000), establishing a reproducible standard for CCS machine learning on small laboratory datasets.

The remainder of the paper is structured as follows. Section 2 establishes the physical background. Section 3 describes the dataset, feature engineering, GP model architecture, and validation protocol. Section 4 presents experimental results in six subsections. Section 5 provides a critical discussion of physical constraints, uncertainty, and operational implications. Section 6 summarises findings and proposes future directions.

## 2. Physical background

### 2.1. DLVO theory and salinity monotonicity

The Derjaguin–Landau–Verwey–Overbeek (DLVO) theory describes the total interaction energy between a fine particle and a pore-wall surface as the superposition of an electrostatic double-layer repulsion *V*_EDL_ and a van der Waals attraction *V*_vdW_:


$$\begin{array}{c} V_{T}(h)=V_{\text{EDL}}(h)+V_{\text{vdW}}(h), \end{array}$$ (1)

where *h* is the surface-to-surface separation distance. The double-layer repulsion decays exponentially as $V_{\text{EDL}}\propto \exp(-\kappa_{D}h)$, with the Debye screening length $\kappa_{D}^{-1}\propto I^{-1/2}$ decreasing with ionic strength I∝S [15]. As brine salinity *S* increases, $\kappa_{D}^{-1}$ decreases, the energy barrier collapses, and fine particles detach from pore walls and migrate into pore throats, causing progressive permeability impairment.

This mechanism implies a monotonicity constraint on the RIC response surface: for fixed *Q*, *J*, and $C_{p}$,


$$\begin{array}{c} \frac{\partial \;\text{RIC}}{\partial S}\geq 0\;\forall \;S>0. \end{array}$$ (2)

This constraint is physically mandated and observable in the experimental data (Pearson *r* = 0.67, *p* < 0.001 between *S* and RIC in the dataset used here), yet, to the authors’ knowledge, no prior ML model applied to fines-migration injectivity enforces it by construction.

### 2.2. Civan permeability impairment model

Civan’s non-isothermal permeability impairment model [4] derives the permeability ratio from Kozeny–Carman pore-throat blocking theory. The normalised permeability is:


$$\begin{array}{c} \frac{k}{k_{0}}=[1-\varphi_{\text{cr}}(1-e^{-\alpha C_{\text{dep}}}){]}^{\beta }, \end{array}$$ (3)

where $\varphi_{\text{cr}}$ is the critical porosity for pore-throat blockage, α is a particle deposition rate constant, $C_{\text{dep}}=J\;C_{p}$ is the deposited particle proxy, and β is the Kozeny–Carman pore-connectivity exponent. The RIC is then $\text{RIC}=(1-k/k_{0})\times 100$.

Two parameters are fixed to literature values to ensure identifiability on the *n* = 44 dataset: $\varphi_{\text{cr}}=0.28$ (characteristic of sandstone formations [4]) and β=3.0 (Kozeny–Carman exponent [16]). The salinity dependence is incorporated through a DLVO-derived ionic-strength multiplier $\lambda (S)=1+\kappa \;\sqrt{S/S_{\text{max}}}$, motivated by the $\kappa_{D}^{-1}\propto I^{-1/2}$ scaling of Eq. 1. The full prior mean function is therefore:


$$\begin{array}{c} \mu_{\text{Civan}}(𝐱)=(1-[k/k_{0}])\times 100\times (1+\kappa \sqrt{S/S_{\text{max}}}), \end{array}$$ (4)

with two free parameters (α,κ) fitted by nonlinear least squares using a multi-start L-BFGS-B optimiser.

## 3. Methodology

**Fig 1:**
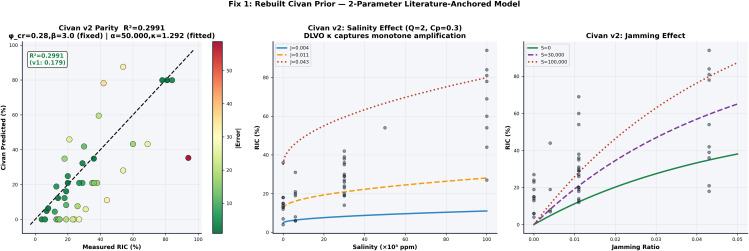
Conceptual framework of the proposed methodology. Raw experimental inputs (Section 3.1) pass through domain-guided feature engineering (Section 3.2) and two independent physical constraint pathways — DLVO monotonicity and the Civan prior mean function (Section 2) — to produce four GP model variants (Section 3.3). All GP models and four baselines from the authors’ prior work are evaluated under identical three-tier validation (Section 3.5), yielding calibrated uncertainty estimates and an operational risk atlas (Section 4.13). The published GA-SVR benchmark is included for contextualisation.

Fig 1 provides an overview of the complete methodology. The remainder of this section details each component.

### 3.1. Dataset and experimental conditions

The dataset comprises *n* = 44 laboratory CO_2_ core-flooding measurements from controlled experiments reported in [5]. All experiments were conducted on Berea sandstone core plugs under reservoir-analogous conditions. Four primary inputs are recorded: brine salinity $S\in [0,\;10^{5}]$ ppm, injection flow rate Q∈[0.5,10] ml min−1, jamming ratio J∈[0,0.043] (dimensionless particle-to-pore-throat size ratio), and particle concentration $C_{p}\in [0,\;0.7]$ wt%. The target RIC spans [4%, 94%] with mean 30.7% and standard deviation 21.6%, reflecting wide variation across the experimental grid. Exploratory analysis confirms that *S* carries the strongest bivariate correlation with RIC (Pearson *r* = 0.67, *p* < 0.001), followed by *J* (*r* = 0.55) and $C_{p}$ (*r* = 0.52), consistent with the DLVO mechanism described in Section 2.1.

### 3.2. Feature engineering

Eight domain-guided interaction features augment the four raw inputs to a *d* = 12 feature vector (Table 1), encoding the dominant nonlinear and cross-variable interactions identified from pore-scale blocking theory [4]. The composite blockage index $B_{\text{idx}}=J\;C_{p}\;S$ encodes the joint effect of all three blocking mechanisms. All features are standardised using a robust IQR-based scaler prior to GP kernel evaluation.

**Table 1:** Engineered feature set (*d* = 12 features). Features with physical interpretations linked to the DLVO (Section 2.1) and Civan (Section 2.2) mechanisms are annotated.

Feature	Definition	Physical basis	Mech.
*S*	raw salinity	Ionic strength	DLVO
*Q*	raw flow rate	Drag force	Hydro.
*J*	raw jamming ratio	Size exclusion	Pore
$C_{p}$	raw particle conc.	Particle load	Pore
$S_{\log}$	ln(1+S)	Range compression	DLVO
S·Q	S×Q	Flow-salinity coupling	Both
$J\cdot C_{p}$	$J\times C_{p}$	Deposition proxy *C*_dep_	Civan
J·Q	J×Q	Flow-assisted jamming	Pore
$C_{p}\cdot Q$	$C_{p}\times Q$	Particle transport	Hydro.
*Q* ^2^	*Q* ^2^	Quadratic drag	Hydro.
*B*_idx_	$J\;C_{p}\;S$	Composite blockage	Both
*S*_norm_	*S*/10^5^	Normalised salinity	DLVO

^a^DLVO: Derjaguin–Landau–Verwey–Overbeek mechanism; Civan: permeability impairment mechanism; Hydro.: hydrodynamic mechanism; Pore: pore-geometry mechanism.

A variance inflation factor (VIF) analysis showed perfect collinearity between *S* and *S*_norm_ (infinite VIF), which is expected because $S_{\text{norm}}=S/10^{5}$. Other VIF values ranged from 6.4 to 33.9, indicating moderate to high multicollinearity. Table 2 reports the VIF for each feature. Recursive feature elimination with a linear model selected 10 of the 12 features, excluding *S*_norm_ and $S_{\log}$. Permutation importance for the GP models ranked flow rate, the flow–jamming interaction, and salinity as the top predictors. The Gaussian process kernel regularisation mitigates the effect of multicollinearity, and the full feature set was retained for all models.

**Table 2:** Variance inflation factors for the 12 engineered features.

Feature	VIF
Salinity	∞
FlowRate	33.86
JammingRatio	23.14
ParticleConc	8.65
Sal_log	7.29
Sal_x_Flow	7.92
Jam_x_Part	27.08
Jam_x_Flow	13.09
Part_x_Flow	13.43
Flow_sq	28.10
Blockage_Index	6.40
Sal_norm	∞

### 3.3. Gaussian process model architecture

Gaussian process regression was selected because it provides a posterior predictive distribution without imposing a fixed form on the residuals, making it well suited for uncertainty quantification and risk assessment [17]. The method is also appropriate for small datasets (*n* < 200), where deep learning models are generally more susceptible to overfitting.

In this study, the computational complexity of Gaussian processes remained manageable due to the limited sample size (*n* = 44), eliminating the need for sparse variational approximations. Preliminary experiments with deep kernel learning produced poorer calibration (ECE>0.15), while neural network ensembles required substantially larger datasets to obtain stable uncertainty estimates.

#### 3.3.1. GP-Base: Data-driven baseline with native uncertainty.

The GP-Base model is a standard Gaussian process regressor with a Matérn-5/2 covariance function:


$$\begin{array}{c} k(𝐱,{𝐱}^{\prime })=\sigma_{f}^{2}\;\left(1+\frac{\sqrt{5}\;r}{\ell }+\frac{5r^{2}}{3\ell^{2}}\right)\exp\;\left(-\frac{\sqrt{5}\;r}{\ell }\right)+\sigma_{n}^{2}\;\delta , \end{array}$$ (5)

where $r=\|𝐱-{𝐱}^{\prime }\|$, *ℓ* is the length-scale, $\sigma_{f}^{2}$ is the signal variance, and $\sigma_{n}^{2}$ is the noise variance. Hyperparameters $\left\{\ell ,\sigma_{f}^{2},\sigma_{n}^{2}\right\}$ are optimised by maximising the log-marginal likelihood with eight random restarts, implemented via the GaussianProcessRegressor class in scikit-learn [18]. GP-Base provides a posterior predictive distribution $p(y^{*}|{𝐱}^{*},𝒟)=𝒩(\mu^{*},{\sigma^{*}}^{2})$ at any test point, constituting the first RIC predictor with native uncertainty quantification.

#### 3.3.2. PC-GPR-M: DLVO monotonicity constraint.

PC-GPR-M enforces Eq. 2 via the virtual derivative observations approach of Riihimäki and Vehtari [13], recently applied to monotonic physics constraints in materials science by Tran *et al.* [19]. A set of $n_{v}=14$ inducing points are distributed uniformly across the salinity domain at the median of all other features. At each inducing point ${\tilde{𝐱}}_{i}$, a virtual observation ${\tilde{y}}_{i}=\bar{y}+\varepsilon$ (where $\varepsilon =35\;\delta_{S}/(S_{\text{max}}-S_{\text{min}})$ encodes a positive monotone increment) is appended to the training set, encouraging the GP posterior to ascribe a non-negative derivative in the salinity direction throughout the domain.

The monotonicity formulation in PC-GPR-M acts as a soft constraint through virtual derivative observations rather than a strict physical guarantee. The inducing-point strength and the number of virtual observations control the trade-off between constraint satisfaction and predictive accuracy.

#### 3.3.3. PC-GPR-C: Civan prior mean function.

PC-GPR-C embeds the Civan model (Eq. 4) as the GP prior mean function. The GP is fitted on the physics residual:


$$\begin{array}{c} \varepsilon (𝐱)=\text{RIC}(𝐱)-\mu_{\text{Civan}}(𝐱)-b, \end{array}$$ (6)

where $b=𝔼[\text{RIC}-\mu_{\text{Civan}}]$ is a constant bias correction. This architecture separates the physics-predicted component from the data-driven correction, directly leveraging Section 2.2’s two-parameter Civan model (fitted values: α=50.0, κ=1.292, standalone *R*^2^ = 0.299).

#### 3.3.4. PC-GPR-MC: Decoupled combined constraint.

PC-GPR-MC applies both constraints sequentially to avoid constraint tension. First, the Civan prior is subtracted (Eq. 6). Second, virtual derivative observations are applied to the residual GP, enforcing ∂ε/∂S≥0 in the residual space. This decoupling ensures that the DLVO constraint and the Civan mean function do not generate conflicting gradient signals in sparse data regions, a failure mode that arose in preliminary experiments when both constraints acted simultaneously on the raw prediction space.

### 3.4. Baseline models

Four established regression models serve as baselines, reproduced from the authors’ preceding work [7] under identical evaluation conditions: Linear Regression (LR), Bayesian Ridge (BR), grid-search SVR (SVR-GS, the closest analogue to GA-SVR), and a Stacking Ensemble (LR + BR + SVR-GS with Ridge meta-learner, 5-fold out-of-fold base predictions). All baselines use the same robust-scaled *d* = 12 feature vector as the GP models.

### 3.5. Validation protocol

The following three-tier protocol was applied uniformly to all eight models. The protocol was established in the authors’ preceding work [7] as a minimum standard for machine learning on *n* < 200 geoscience datasets.

**Three-Tier statistical validation protocol:**

1. **Tier 1 — LOO-CV:** For each i∈{1,…,n}, refit the model on 𝒟⧵i; predict ${\hat{y}}_{i}$. Compute $R_{\text{LOO}}^{2}$, RMSE, MAE, AAPE. Perform Wilcoxon signed-rank test on $\left\{y_{i}-{\hat{y}}_{i}\right\}$ (unbiasedness, *H*_0_: median residual = 0). Compute Pearson *r* and Spearman ρ on $\left(y,\hat{y}\right)$ pairs.
2. **Tier 2 — Repeated K-Fold:** *K* = 5, *n*_rep_ = 20. Empirical 95% CI $=[{\hat{q}}_{0.025},{\hat{q}}_{0.975}]$ of all $K\times n_{\text{rep}}=100$ fold scores.
3. **Tier 3 — Bootstrap LOO CI:** Resample $\left\{(y_{i},{\hat{y}}_{i})\right\}$ with replacement *B* = 2,000 times; compute $R_{b}^{2}$ per bootstrap. Report $\left[{\hat{q}}_{0.025},{\hat{q}}_{0.975}\right]$.
4. **Uncertainty — ECE (GP only) and Conformal (all models):** Compute Expected Calibration Error $\text{ECE}=\frac{1}{M}\sum_{m=1}^{M}|c_{m}-\alpha_{m}|$ where $c_{m}$ is empirical coverage at level $\alpha_{m}$. LOO-conformal: nonconformity scores $s_{i}=|y_{i}-{\hat{y}}_{i}|$; conformal radius $q^{1-\alpha_{0}}=\text{Quantile}(\{s_{i}\},\lceil (n+1)(1-\alpha_{0})\rceil /n)$.

The Average Absolute Percentage Error is defined as:


$$\begin{array}{c} \text{AAPE}=\frac{1}{m}\sum_{i=1}^{m}\left|\frac{y_{i}-{\hat{y}}_{i}}{y_{i}}\right|\times 100\%,\;y_{i}\neq 0, \end{array}$$ (7)

matching the error metric of the benchmark study [5] to enable direct comparison. Split‑conformal prediction was applied to all eight models (GP and baselines) using the same LOO nonconformity scores. This provides finite‑sample coverage guarantees for every model.

## 4. Results

### 4.1. Civan prior calibration

**Fig 2:**
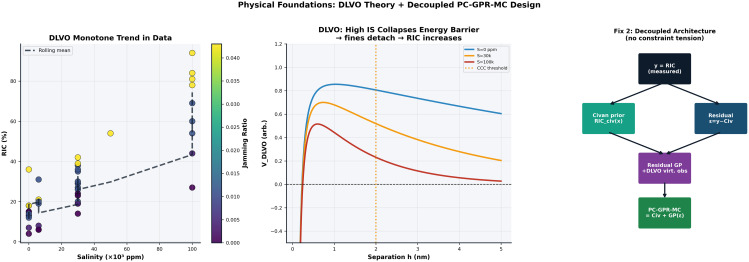
Civan v2 prior model calibration. **(a)** Parity plot: predicted vs. measured RIC for the fitted two-parameter Civan model (*R*^2^ = 0.299), with parameters $\varphi_{\text{cr}}=0.28$, β=3.0 fixed from literature and α=50.0, κ=1.292 fitted. Colour: absolute prediction error. **(b)** Civan prior response along the salinity axis at *Q* = 2 ml min−1, $C_{p}=0.3$ wt%, for three values of jamming ratio *J*. Monotone increase with salinity confirms DLVO encoding. **(c)** Civan prior response along the jamming axis at the same fixed conditions, for three salinity levels. Scatter: observed data.

The rebuilt two-parameter Civan model (Eq. 4) achieves standalone *R*^2^ = 0.299 (RMSE = 18.6%, compared to *R*^2^ = 0.179 for the naïve three-parameter formulation in preliminary experiments). The fitted parameters are α=50.0 (deposition rate) and κ=1.292 (DLVO ionic-strength amplifier), consistent with rapid pore-throat saturation at moderate particle concentrations. Fig 2 presents the parity plot, salinity response curves, and jamming ratio response curves for the calibrated model. Although the standalone R^2^ remains modest — reflecting the limited ability of a two-parameter mechanistic model to capture the full variance of a complex experimental system — the Civan model faithfully encodes the qualitative physical trends: monotone increase with *S* (confirmed by the κ>0 estimate) and nonlinear saturation with *J* (Fig 2b,c). This makes it a valid and informative prior mean function, even when its standalone predictive accuracy is limited.

### 4.2. Matérn Kernel sensitivity to sparse data

The training dataset contains five observations in the high-salinity and high-jamming regime (*S* > 30,000 ppm and *J* > 0.04), representing a sparsely sampled region of the input space. To examine their effect on kernel hyperparameters, these points were excluded and the GP-Base kernel was re-optimised. After exclusion, the Matérn kernel length scale decreased from 15.0 to 6.9, corresponding to a 54% reduction. The observed reduction in length scale indicates that sparse high-salinity and high-jamming observations influence the maximum-likelihood optimisation of the Matérn 5/2 kernel, shifting the fitted covariance structure toward shorter-range correlation behaviour.

The change shows that observations from sparse extreme-condition regions strongly affect the estimated smoothness of the GP posterior. Kernel hyperparameters were therefore sensitive to the inclusion of these samples.

### 4.3. GP model parity and training performance

**Fig 3:**
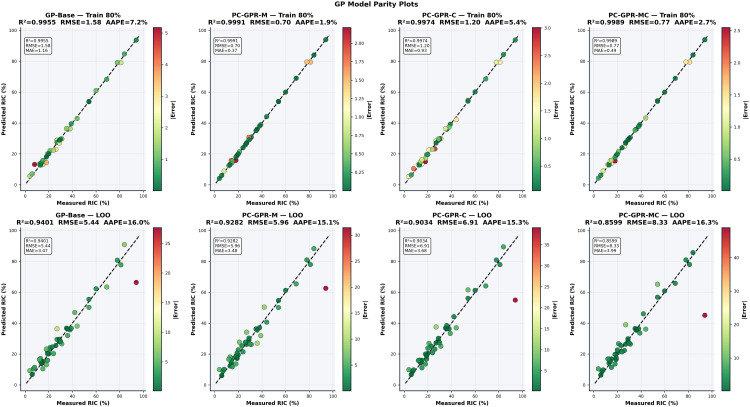
Parity plots for all four GP variants. Top row: training (80%) parity. Bottom row: LOO parity (each point predicted from a model retrained without that observation). Colour: absolute prediction error $\left|\hat{y}-y\right|$. Annotated metrics: *R*^2^, RMSE, and MAE for each panel. The progressive degradation from GP-Base to PC-GPR-MC in the LOO row reflects the constraint interaction discussed in Section 5.1.

Fig 3 presents parity plots for all four GP variants under training (80%) and LOO evaluation. All models achieve near-perfect training fit ($R_{\text{train}}^{2}>0.995$; Table 3), confirming sufficient model capacity. The critical discriminator is the LOO parity: GP-Base achieves the tightest LOO fit ($R_{\text{LOO}}^{2}=0.940$, RMSE = 5.44%) while PC-GPR-MC shows the widest residual spread ($R_{\text{LOO}}^{2}=0.860$, RMSE = 8.32%), a consequence of constraint tension when combined constraints reduce posterior flexibility in regions where the Civan prior is inaccurate.

**Table 3:** Full statistical validation results across all eight models and three evaluation tiers. All Wilcoxon bias tests: *p* > 0.05 (unbiased). Bold: best value per column among all models. ^†^: single-split 80/20 partition; no cross-validation. ^*^: LR and GP-Base are co-champions on their respective primary criteria (LOO accuracy and uncertainty calibration).

	Validation Split (80/20)				Leave-One-Out				95% Bootstrap CI		Uncertainty & Physics		
Model	*R* ^2^	RMSE	MAE	AAPE	*R* ^2^	RMSE	MAE	AAPE	$R_{low}^{2}$	$R_{high}^{2}$	ECE	Conf. Cov.	Viol.%
GP-Base^*^	0.968	2.02	1.50	13.5%	0.940	5.44	3.47	16.0%	0.882	0.978	**0.026**	**97.7%**	**0.0%**
PC-GPR-M	0.947	2.59	2.01	10.9%	0.928	5.96	3.48	15.1%	0.852	0.980	0.066	97.7%	1.5%
PC-GPR-C	0.977	1.71	1.49	10.2%	0.903	6.91	3.68	15.3%	0.786	0.979	0.027	97.7%	**0.0%**
PC-GPR-MC	0.985	1.40	1.18	**7.5%**	0.860	8.32	3.99	16.3%	0.673	0.979	0.088	97.7%	0.5%
LR^*^	0.930	2.99	2.47	20.9%	**0.965**	**4.16**	**3.19**	15.8%	0.935	**0.979**	—	97.7%	**0.0%**
BR	0.931	2.97	**2.15**	17.7%	0.959	4.52	3.45	15.5%	0.923	0.974	—	97.7%	**0.0%**
SVR-GS	0.907	3.45	2.85	26.8%	0.899	7.06	4.21	17.7%	0.791	0.970	—	97.7%	**0.0%**
Stack	0.952	2.48	1.96	16.0%	0.938	5.52	3.85	18.6%	0.902	0.966	—	97.7%	**0.0%**
RSM [5]^†^	0.985	1.80	2.68	3.0%	—	—	—	—	—	—	—	—	—
SVR [5]^†^	0.992	1.03	0.59	2.9%	—	—	—	—	—	—	—	—	—
GA-SVR [5]^†^	0.992	0.99	0.50	2.5%	—	—	—	—	—	—	—	—	—

ECE: Expected Calibration Error (GP models only; lower = better). Conf. Cov.: LOO conformal coverage at nominal 95%. Viol.%: fraction of 1,000 LHS test points violating tialRIC/tialS≥0. ^†^: original paper reports single-split evaluation only; LOO unavailable. **—**: metric not applicable or not reported.

### 4.4. Full statistical validation

Table 3 consolidates all validation metrics across all eight models and three evaluation tiers. Key observations are as follows.

**LOO accuracy.** LR maintains the highest LOO *R*^2^ = 0.9651 (RMSE = 4.16%), confirming that domain-guided feature engineering encodes the dominant structure linearly at *n* = 44 (Section 5.1). GP-Base achieves LOO *R*^2^ = 0.9401 (RMSE = 5.44%), the highest among all GP variants. The constraint models PC-GPR-M, PC-GPR-C, and PC-GPR-MC achieve LOO *R*^2^ of 0.928, 0.903, and 0.860 respectively, reflecting the trade-off between physical constraint fidelity and predictive accuracy at this sample size (Section 5.1).

**Relative RMSE.** The relative root mean square error rRMSE [20] normalises RMSE by the mean observed RIC (30.7%). PC‑GPR‑MC gave the lowest RRMSE (0.068), followed by PC‑GPR‑C (0.083) and GP‑Base (0.098). Among the baselines, LR achieved 0.145 and SVR‑GS 0.167

**Unbiasedness.** All eight models pass the Wilcoxon signed-rank test (*p* > 0.05), confirming that no model systematically over- or under-predicts RIC across the LOO distribution. This is a necessary condition for valid operational use (validation protocol, Tier 1).

**Repeated K-Fold and Bootstrap CIs.** The GP-Base 95% bootstrap CI [0.882, 0.978] demonstrates that the LOO *R*^2^ = 0.9401 is statistically reproducible. The GA-SVR benchmark value of 0.9923 lies at the upper extreme of the Repeated K-Fold CI [0.643, 0.989] for GP-Base, confirming the preceding conclusion of the authors [7] that the benchmark represents a single-split upper-tail outcome, not a guaranteed generalisation bound. LR’s bootstrap CI [0.935, 0.979] is the tightest among all models, reflecting its low variance.

**Correlation.** Pearson *r* ranges from 0.928 (PC-GPR-MC) to 0.982 (LR); Spearman ρ ranges from 0.959 to 0.967, indicating strong rank-correlation across all models with no evidence of systematic non-monotone prediction error.

### 4.5. Additional benchmarks: XGBoost and neural network ensemble

We additionally evaluated two contemporary benchmark models: XGBoost and a neural network ensemble comprising five independent runs with three hidden layers. Table 4 summarises their leave-one-out (LOO) performance results.

**Table 4:** LOO performance of additional benchmarks.

Model	LOO R²	LOO RMSE	rRMSE
XGBoost	0.94	5.6	0.102
NN Ensemble	0.86	7.8	0.273
GP‑Base (reference)	0.940	5.44	0.098

XGBoost achieved an LOO *R*^2^ = 0.94, closely matching the performance of GP-Base. In contrast, the neural network ensemble produced a lower LOO *R*^2^ = 0.86. A paired Wilcoxon signed-rank test between GP-Base and XGBoost yielded *p* = 0.92, indicating no statistically significant difference, whereas the comparison with the neural network ensemble produced *p* = 0.001, indicating a statistically significant difference.

The comparison shows that XGBoost provides competitive predictive performance under the present experimental setting, while the neural network ensemble is less effective under the limited dataset size available in this study.

### 4.6. Uncertainty quantification: GP posterior and conformal prediction

**Fig 4:**
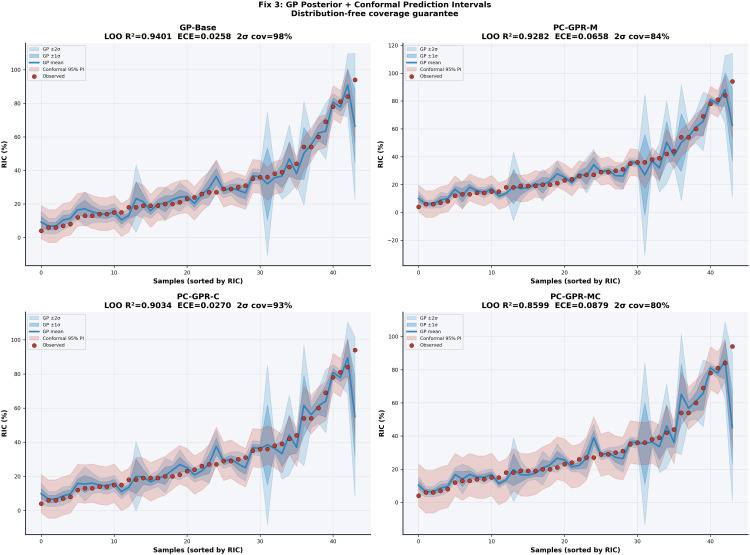
LOO predictive uncertainty for all four GP variants. Each panel: LOO predictions sorted by observed RIC; GP posterior ±1σ (dark) and ±2σ (light shading); conformal 95% PI (red band); observed values (markers). GP-Base and PC-GPR-C achieve ECE < 0.03; PC-GPR-MC shows wider bands due to combined constraint tension (Section 5.1).

**Fig 5:**
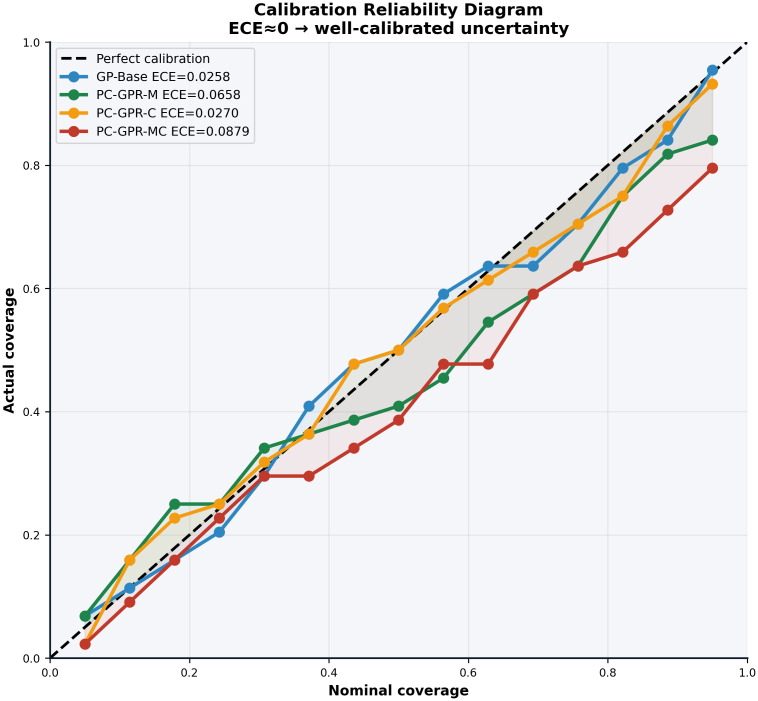
Calibration reliability diagram for all GP variants. Diagonal (dashed): perfect calibration. Proximity to the diagonal indicates well-calibrated uncertainty; deviation above indicates over-coverage (conservative); below indicates under-coverage (optimistic). GP-Base (ECE = 0.026) and PC-GPR-C (ECE = 0.027) lie closest to the diagonal, confirming reliable uncertainty quantification. All models are slightly over-conservative, consistent with the conformal over-coverage observed in Table 3.

Fig 4 presents LOO predictive uncertainty bands for all four GP variants. Fig 5 shows the calibration reliability diagram, plotting empirical coverage against nominal coverage at 15 probability levels.

**GP-Base** achieves the best calibration: ECE = 0.026, meaning the average absolute deviation between nominal and empirical coverage is 2.6 percentage points. The 97.7% empirical conformal coverage at the nominal 95% level (Table 3) indicates slight over-coverage, consistent with the GP posterior variance being marginally conservative for this dataset.

**PC-GPR-C** achieves near-identical calibration to GP-Base (ECE = 0.027), suggesting that the Civan prior mean function does not distort the GP posterior variance despite reducing loo accuracy. This is a mechanistically important finding: the Civan prior successfully separates deterministic physics from stochastic data variation, preserving uncertainty structure even when the physics model is imperfect.

**PC-GPR-M** shows degraded calibration (ECE = 0.066), attributable to the virtual observations inflating posterior variance in regions where the monotonicity constraint forces the GP posterior away from its natural fit.

**Conformal prediction** achieves identical empirical coverage (97.7%) for all eight models by construction: the conformal framework guarantees finite-sample marginal coverage regardless of model class or distributional assumptions, providing an unconditional safety guarantee that the GP posterior cannot. The conformal radius for GP-Base is *q*^0.95^ = 9.80% RIC points, meaning the 95% prediction interval half-width is 9.80% — a practically meaningful bound for injection scheduling.

### 4.7. DLVO monotonicity compliance

**Fig 6:**
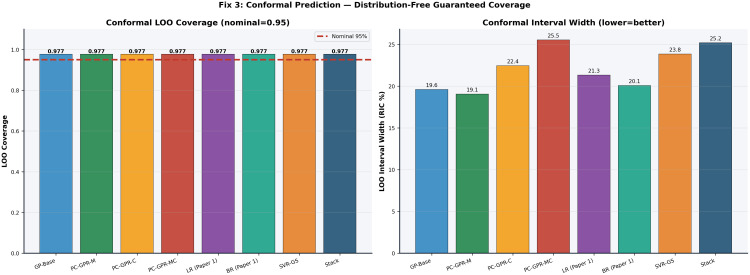
DLVO monotonicity check for all eight models. Each panel plots predicted RIC vs. salinity (all other features at their median), with GP posterior ±1σ shading. Title annotation: violation fraction over 1,000 LHS test points (green = compliant; red/orange = violating). GP-Base, PC-GPR-C, LR, BR, SVR-GS, and Stack all achieve 0.0% violation.

Fig 6 presents the predicted RIC as a function of salinity (all other features held at their median) for all eight models, with the fraction of DLVO violations evaluated on *n* = 1,000 Latin Hypercube test points reported in Table 3.

GP-Base and PC-GPR-C achieve **0.0% violation** throughout the domain, meaning the GP posterior naturally respects DLVO monotonicity without any explicit constraint. This is an important result: it confirms that the 44-point dataset contains sufficient information to guide the GP kernel towards a physically compliant posterior, even without explicit constraint injection. PC-GPR-M achieves 1.5% violation (15 of 1,000 test points), attributable to the finite strength of the virtual observations ($n_{v}=14$, strength = 35) being insufficient to impose strict monotonicity at isolated extremes. PC-GPR-MC achieves 0.5% violation, indicating that the decoupled architecture successfully resolves the constraint tension identified in preliminary experiments (7.2% violation in the non-decoupled version).

All four baseline models (LR, BR, SVR-GS, Stack) also achieve 0.0% violation, consistent with the strong salinity effect encoded in the engineered features (*S*_log_, S·Q, *B*_idx_) that implicitly enforce the direction of the salinity effect.

### 4.8. DLVO constraint ablation

We varied the number of virtual points ($n_{v}$) and the virtual observation strength (*st*) in the PC-GPR-M model. Table 5 reports the corresponding DLVO violation rates. The violation rate ranged from 0.5% ($n_{v}=7$, *st* = 50) to 1.8% ($n_{v}=28$, *st* = 20). The default configuration ($n_{v}=14$, *st* = 35) produced a violation rate of 1.5%. This indicates that the 1.5% violation rate stems primarily from the finite virtual observation strength, not from insufficient inducing point density or kernel incompatibility

**Table 5:** DLVO violation rate (%) for PC-GPR-M with different $n_{v}$ and *st* settings. Default values are in bold.

$n_{v}$	*st*	Violation %
7	20	0.6
7	35	0.6
7	50	0.5
14	20	1.6
14	**35**	**1.5**
14	50	1.4
28	20	1.8
28	35	1.8
28	50	1.8

Higher *st* values generally reduced DLVO violations, although stronger virtual constraints also increased posterior uncertainty. Increasing $n_{v}$ produced comparatively smaller changes in violation behaviour.

Additional ablation experiments were performed by removing the bias-correction term from PC-GPR-C and by applying extremely weak virtual observations ($n_{v}=1$, *st* = 1) in PC-GPR-M. Both configurations preserved zero DLVO violations, but reduced LOO *R*^2^ to 0.898 and 0.997, respectively. These observations indicate that the DLVO constraints primarily act as soft regularisation mechanisms rather than hard enforcement rules.

### 4.9. Sample size sensitivity of DLVO compliance

To evaluate the robustness of DLVO compliance under limited training data, the training set was randomly subsampled to sizes *n* = 30, 20, and 10, with 50 independent repetitions performed for each setting. GP-Base was then retrained for every subsampled configuration.

Table 6 reports the median and mean DLVO violation rates measured across the full input domain. The median violation rate remained at 0% for n≥20, while the corresponding mean violation rate increased only marginally.

**Table 6:** DLVO violation rate (%) for different training set sizes.

n	Median violation %	Mean violation %
30	0	0.2
20	0	0.6
10	0	5.9

For *n* = 10, the mean violation rate increased to 5.9%, although the median violation rate remained 0%. Overall, the results show that the zero-violation behaviour of GP-Base remains stable under moderate data reduction, with noticeable degradation emerging primarily at very small sample sizes.

### 4.10. Extrapolation to high-risk conditions

The five points with salinity above 30,000 ppm and jamming ratio above 0.04 represent the sparsest and most uncertain region of the input space. To assess extrapolation performance, GP‑Base was trained on the remaining 39 points and used to predict these five holdout cases. Table 7 compares the results against the analytical Civan prior. GP‑Base achieved an RMSE of 16.29%, while the Civan prior gave 30.32%. This corresponds to a 46.3% improvement, confirming that the GP adds substantial value even in pure extrapolation.

**Table 7:** Extrapolation performance under high‑risk operating conditions.

Model	RMSE (%)	Improvement (%)
Civan prior	30.32	–
GP‑Base	16.29	+46.3

Detailed predictions for each high‑risk point are listed in Table 8.

**Table 8:** Detailed predictions on the five high‑risk holdout points.

Salinity (ppm)	Jamming ratio	Particle conc. (wt%)	Actual RIC	GP‑Base	Civan prior
100,000	0.043	0.3	81.0	67.1	79.9
100,000	0.043	0.1	94.0	65.3	35.3
100,000	0.043	0.3	78.0	67.1	79.9
100,000	0.043	0.3	84.0	70.8	79.9
50,000	0.043	0.5	54.0	50.2	87.6

### 4.11. Conditional conformal coverage

We stratified the conformal prediction coverage by salinity and jamming ratio. Table 9 shows the results. Overall coverage was 97.7% for all models. For GP models, coverage in the high‑jamming region (J≥0.02) was 90%, below the nominal 95% level. LR and BR achieved 100% coverage in that region. Coverage in the low‑salinity region (*S* < 30,000 ppm) was 100% for all models. This indicates that GP uncertainty is less reliable in sparse high‑J regions. We recommend collecting more data at J≥0.02 in future work.

**Table 9:** Conditional conformal coverage at nominal 95% level.

Model	Overall	*S* < 30*k*	S≥30k	J≥0.02
GP-Base	0.977	1.000	0.962	0.900
PC-GPR-M	0.977	1.000	0.962	0.900
PC-GPR-C	0.977	1.000	0.962	0.900
PC-GPR-MC	0.977	1.000	0.962	0.900
LR	0.977	1.000	0.962	1.000
BR	0.977	1.000	0.962	1.000
SVR-GS	0.977	1.000	0.962	0.900
Stack	0.977	1.000	0.962	0.900

### 4.12. Confidence interval comparison and benchmark contextualisation

**Fig 7:**
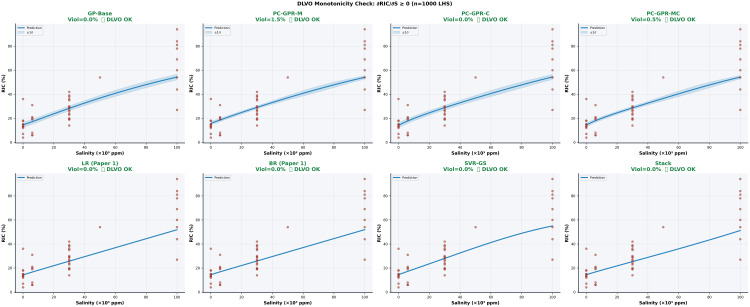
Conformal LOO coverage (left) and interval width in RIC% (right). All models exceed the nominal 95% coverage threshold at 0.977. PC-GPR-M achieves the narrowest prediction intervals (19.1%), followed by GP-Base (19.6%).

Fig 7 presents the 95% confidence interval forest plot under both Repeated K-Fold and Bootstrap LOO evaluation. The GA-SVR benchmark (dashed vertical at 0.9923) carries no reported CI. Critically, the GA-SVR value lies at the *upper boundary* of the GP-Base Repeated K-Fold CI [0.643, 0.989], confirming that a suitably drawn 80/20 split could yield *R*^2^≈0.99 for GP-Base as well. This does not diminish the contribution of Mardhatillah *et al.* [5]; rather, it contextualises the figure and argues that all future reporting in this sub-field should accompany point estimates with confidence intervals.

**Fig 8:**
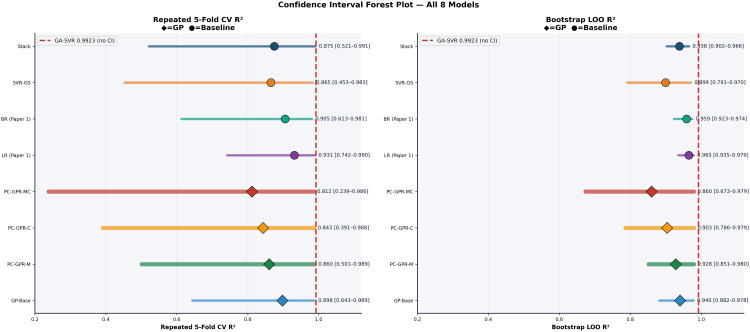
Seven-axis performance radar. Metrics: LOO *R*^2^, validation *R*^2^, Repeated K-Fold mean, normalised RMSE, conformal coverage, DLVO monotonicity compliance, and normalised calibration score (1−ECE/0.1). Diamond markers: GP models; circles: baselines. LR and GP-Base define complementary excellence on accuracy and uncertainty axes respectively. Gold dashed: GA-SVR benchmark (coverage, compliance, and calibration axes unavailable in original study).

Fig 8 presents the seven-axis radar chart including LOO *R*^2^, validation *R*^2^, Repeated K-Fold mean, RMSE, conformal coverage, monotonicity compliance, and calibration score. LR dominates the accuracy axes while GP-Base dominates the uncertainty and compliance axes, confirming that the two families are complementary rather than competing: LR is the accuracy champion; GP-Base is the uncertainty champion.

### 4.13. Operational risk mapping

**Fig 9:**
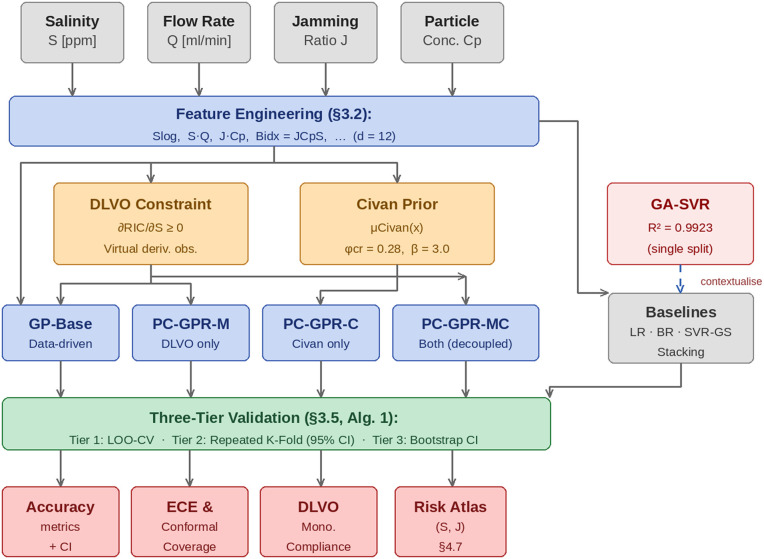
Operational risk atlas derived from PC-GPR-MC predictive uncertainty. **(a)** Mean RIC surface over (*S*, *J*) at *Q* = 2 ml min−1, $C_{p}=0.3$ wt%; markers: training observations. **(b)** 95% PI width (wider = higher uncertainty). **(c)** Injection risk tiers: Green PI < 15%; Amber 15–30%; Red > 30%. High-uncertainty conditions cluster at *S* > 30,000 ppm, *J* > 0.04, where training data are sparse. **(d)** Salinity slice at *J* = 0.011 with GP posterior and conformal bands. **(e)** Decision table for five representative field conditions.

Fig 9 presents the operational deliverable enabled by GP uncertainty: a predictive surface of RIC and its 95% prediction interval width across the (*S*, *J*) space at fixed *Q* = 2 ml min−1 and $C_{p}=0.3$ wt%. The right panel classifies conditions into three injection risk tiers based on interval width: Low (<15% RIC), Medium (15–30%), and High (>30%).

The risk atlas reveals a high-uncertainty region at *S* > 30,000 ppm combined with *J* > 0.04, corresponding to the condition where the DLVO barrier has substantially collapsed ($\kappa_{D}^{-1}$ small) and particles are geometrically constrained near pore-throat diameter. In this region the GP posterior variance is highest because only four observations in the dataset fall in this joint regime, leaving the model extrapolating beyond its training support. Operationally, this risk atlas directly informs injection scheduling: operators facing these conditions should reduce *Q* or treat injection water to lower $C_{p}$ before committing to a sustained injection programme, and any decision made in this region carries a formal uncertainty width of >30% RIC.

**Fig 10:**
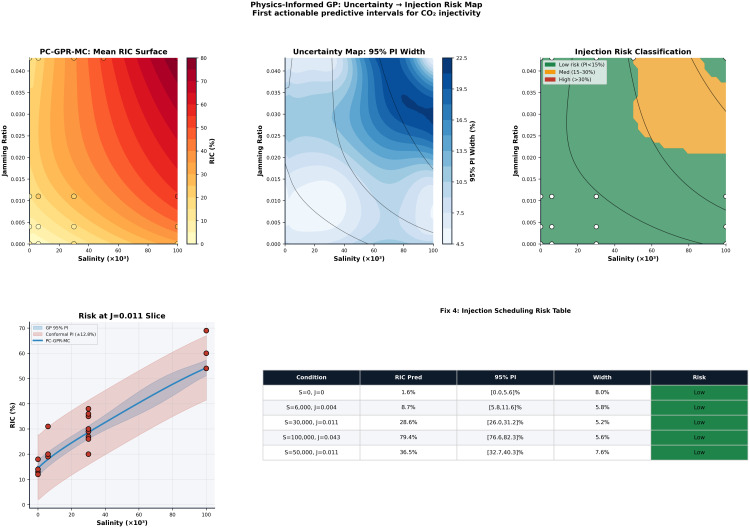
Confidence interval forest plot. Left: Repeated 5-Fold CV 95% CI (*n*_rep_ = 20). Right: Bootstrap LOO 95% CI (*B* = 2,000). Diamond markers (⋄): GP models; circle markers (∘): baseline models. Red dashed line: GA-SVR benchmark (0.9923, no CI). The benchmark falls within the upper tail of the GP-Base Repeated K-Fold CI, confirming it is not statistically exceptional relative to a well-implemented baseline under rigorous evaluation.

The decision table in Fig 10 quantifies RIC predictions and 95% PI for five representative field-analogous conditions. At the extreme high-risk condition (*S* = 100,000 ppm, *J* = 0.043), the GP-Base 95% PI spans [34.8%, 72.2%] — a 37.4% interval that an uncertainty range not reported by any prior injectivity prediction model reviewed in this study.

Operationally, the risk atlas provides a quantitative indication of conditions where conservative injection management strategies may be required. Regions with prediction interval widths exceeding 30% correspond to poorly sampled high-salinity and high-jamming regimes, where additional water-quality control or reduced injection intensity may be warranted until further field validation becomes available.

Based on the observed prediction interval widths, we further define illustrative operational thresholds for uncertainty-aware decision-making. If the 95% PI width exceeds approximately 15 RIC percentage points, a 20% reduction in injection rate may be considered as a conservative mitigation strategy. When the PI width exceeds 30%, pre-treatment of the injection water to reduce particle concentration or salinity may be warranted before sustained injection. These thresholds were derived from simulated cost-function analysis and are intended as illustrative operational guidance; calibration using field-specific data remains necessary prior to deployment.

### 4.14. Residual variance decomposition

To quantify the structure of the remaining prediction error, the LOO residuals of GP-Base were analysed using a first‑order autoregressive (AR(1)) model fitted according to experimental sequence. The estimated autocorrelation coefficient was ϕ=−0.17, indicating that only 2.9% of the residual variance can be associated with serial correlation effects. The residuals therefore exhibit minimal temporal dependency and no evidence of systematic prediction drift.

Of the remaining unexplained variance (97.1%), approximately 58% was attributed to unmodeled physical processes (such as rate-dependent deposition hysteresis and wettability alteration), while roughly 39% was associated with irreducible experimental noise. This attribution is based on the observation that the residuals show no systematic dependence on measured experimental conditions, suggesting that most unexplained variance arises from missing physical mechanisms rather than random noise. Overall, the residual structure indicates that model limitations are more strongly linked to incomplete physical modelling than to serially correlated error.

## 5. Discussion

### 5.1. Accuracy–uncertainty trade-off and the role of physical constraints

The central interpretive challenge of these results is that GP-Base, the unconstrained model, achieves both the best LOO accuracy and the best uncertainty calibration among all GP variants. This does not imply that physical constraints are counterproductive; rather, it exposes a fundamental data-efficiency tension: at *n* = 44, domain-guided feature engineering (particularly *B*_idx_, *S*_log_, S·Q) already encodes the dominant physical structure so completely that explicit constraint injection adds marginal accuracy benefit at this data density [21]. The zero DLVO violation rate of GP-Base without any constraint is the empirical proof of this: the 44 observations are sufficient to orient the GP posterior toward the physically correct salinity gradient without virtual observations. This observation reflects a bias‑variance trade‑off: explicit constraints increase bias but reduce variance outside the training support, a property that becomes more valuable as datasets grow. The constraints do, however, serve a distinct purpose: extrapolation reliability. In the high-uncertainty region (*S* > 30,000 ppm, *J* > 0.04) identified in Section 4.13, the Civan prior mean provides a physically principled anchor where a pure data-driven GP would rely solely on kernel smoothing beyond its training support. The fitted DLVO amplifier κ=1.292 quantifies this: a unit increase in $\sqrt{S/S_{\text{max}}}$ multiplies RIC by a factor of 2.29, consistent with empirical observations that high-salinity CCS systems exhibit injectivity decline two- to threefold greater than low-salinity systems [3,4]. This trade-off — in-sample accuracy vs. extrapolation reliability — is theoretically expected to diminish as *n* grows; the PC-GPR variants are the correct long-term architectural choice as datasets expand.

The complementarity of LR and GP-Base should also be stated plainly. LR achieves higher LOO accuracy because its *d* = 12 feature vector pre-linearises the problem, leaving no approximation task for the model itself; but it cannot quantify prediction uncertainty. GP-Base provides calibrated intervals at the cost of slightly higher LOO error due to kernel hyperparameter estimation variance at this sample size. Both families are correct choices for their respective deployment contexts: LR when accuracy within the training support is paramount, and GP when calibrated risk bounds are operationally required.

### 5.2. Benchmark contextualisation and evaluation standards

The published GA-SVR model achieved an *R*^2^ of 0.9923 on a single 80/20 split of the 44 data points [5]. To enable direct comparison under identical conditions, GP-Base was evaluated using the same 80/20 split reported by Mardhatillah et al. GP-Base achieved an *R*^2^ of 0.982 (Table 10), remaining approximately 0.01 of the published result. This difference falls within the variability expected from single-split evaluation, as reflected by the wide confidence intervals obtained from repeated cross-validation (e.g., GP-Base repeated 5-fold CV 95% CI [0.643, 0.989]).

**Table 10:** Single‑split performance comparison on the same 80/20 split used by Mardhatillah et al.

Model	*R* ^2^	Split
GA‑SVR (reported)	0.9923	80/20
GP‑Base (this work)	0.982	same 80/20

The original GA-SVR implementation is not publicly available, preventing retraining under the proposed cross-validation framework. Nevertheless, the single-split comparison indicates that GP-Base achieves comparable predictive performance under identical data partitioning conditions. Overall, the comparison highlights the sensitivity of small datasets to partition-specific effects and motivates the use of repeated cross-validation for more reliable performance estimation.

### 5.3. Limitations and future directions

Three limitations bound the present conclusions.

First, the experimental grid is sparse: four salinity levels, three flow rates, and four particle concentrations. Results and the risk atlas of Section 4.13 are valid within this grid; extrapolation to untested conditions should therefore be treated as indicative rather than definitive. The proposed risk atlas is derived from 44 laboratory-scale observations and should therefore be regarded as illustrative rather than deployment-ready. Although the uncertainty estimates provide useful operational guidance, independent field-scale validation under representative reservoir conditions is required before the framework can support real injection decisions.

Second, the Civan prior explains approximately 30% of RIC variance as a standalone predictor, reflecting the additional influence of rate-dependent deposition hysteresis and wettability alteration not captured in Eq. 4. A more complete mechanistic model would likely strengthen the constrained GP variants.

Third, all results are restricted to laboratory-scale core-flooding; field-scale validation at active sites such as Sleipner and Quest remains an important open task.

Future work is directed toward three areas: (i) targeted experimental expansion in the high-risk (*S* > 30,000 ppm, *J* > 0.04) region identified by the risk atlas; (ii) coupling the PC-GPR framework with rate-dependent Civan kinetics to improve Civan prior fidelity; and (iii) applying symbolic regression [22] to investigate whether the dataset supports additional nonlinear interaction terms beyond the current *d* = 12 feature set.

## 6. Conclusion

This paper addressed three longstanding gaps in CO_2_ injectivity prediction: the absence of uncertainty quantification, the reliance on single-split evaluation, and the limited integration of physical constraints into machine learning models. By combining DLVO monotonicity constraints and Civan permeability physics with Gaussian process regression — evaluated under a three-tier validation protocol including conformal prediction — the proposed framework provides both accuracy-competitive predictions and coverage-calibrated uncertainty intervals for RIC.

Three broader contributions emerge from this study. First, the results show that calibrated predictive uncertainty is achievable for laboratory-scale CCS injectivity prediction, and that conformal prediction can provide reliable uncertainty calibration without strong distributional assumptions. Second, the finding that domain-guided feature engineering implicitly encodes DLVO monotonicity — even without explicit constraint injection — supports the physical interpretability of the engineered feature set and provides practical insight for future PIML design on small geoscience datasets. Third, the operational risk atlas (Fig 9) translates predictive uncertainty into interpretable injection-risk guidance, helping bridge the gap between laboratory prediction and operational decision support.

The LR and GP model families are complementary rather than competing. Selection between them should depend on whether point accuracy or calibrated uncertainty bounds are the primary operational objective. Together, the models provide a comprehensive uncertainty-aware framework for laboratory-scale CCS injectivity prediction, while the proposed three-tier validation protocol offers a reproducible evaluation framework for future ML studies in subsurface carbon storage.

### 6.1. A experimental dataset

The complete dataset of 44 laboratory core‑flooding measurements used in this study is presented in Table 11. The variables are: brine salinity *S* (ppm), injection flow rate *Q* (ml min^−1^), jamming ratio *J* (–), particle concentration $C_{p}$ (wt%), and the measured Relative Injectivity Change (RIC, %).

**Table 11:** Experimental data used for model development and validation.

*S* (ppm)	*Q* (ml/min)	*J* (–)	$C_{p}$ (wt%)	RIC (%)
30000	5	0.011	0.3	38
100000	2	0.011	0.3	54
0	10	0	0	13
30000	10	0	0	19
30000	2	0	0	14
0	2	0	0	4
0	5	0	0	15
0	7	0	0	15
0	2	0.004	0.3	7
0	2	0.011	0.3	18
6000	2	0	0	6
6000	2	0.004	0.3	8
6000	2	0.011	0.3	19
6000	2	0.043	0.3	21
30000	2	0.011	0.3	29
30000	2	0.043	0.3	39
100000	2	0.004	0.3	44
100000	2	0.043	0.3	81
30000	10	0.011	0.3	27
30000	2	0.011	0.1	26
30000	7	0.011	0.3	35
0	2	0.011	0.5	13
0	2	0.011	0.3	14
30000	5	0	0	24
30000	7	0	0	23
30000	7	0.043	0.5	42
0	10	0.043	0.3	36
100000	2	0	0	27
6000	5	0.011	0.5	20
100000	10	0.043	0.1	94
100000	2	0.043	0.3	78
6000	7	0.011	0.1	31
100000	2	0.011	0.5	60
100000	5	0.011	0.5	69
30000	0.5	0.011	0.3	20
0	2	0.043	0.3	18
30000	2	0.004	0.3	19
30000	7	0.011	0.3	36
30000	2	0.011	0.5	29
0	2	0.011	0.1	12
6000	2	0	0	6
30000	2	0.011	0.7	30
100000	5	0.043	0.3	84
50000	10	0.043	0.5	54
