# Supplementary material for: Physics-informed gaussian process regression for reproducible and uncertainty-aware CO2 injectivity prediction
Source: PLoS One. 2026 Jul 1;21(7):e0352178. doi: 10.1371/journal.pone.0352178 (PMC13322525; doi:10.1371/journal.pone.0352178)
Supplement: Attachment — Submitted filename: 2Reviwer_response_22May2026.pdf [file pone.0352178.s002.pdf]

Dear Reviewers,

Thank you for your thoughtful and detailed comments. All 21 queries have been addressed. The manuscript has been strengthened with new analyses, additional tables, and clearer writing. Below is our point-by-point response.

Summary of Revisions

Reviewer	Comment	Manuscript location (section / page)
R1-1	Matérn kernel sensitivity to sparse data	Sec. 4.2 / p.10
R1-2	Residual variance decomposition (AR(1))	Sec. 4.14 / pp.21–22
R1-3	Why GP-Base outperforms constrained variants	Sec. 5.1 / p.22
R1-4	DLVO violation cause (ablation)	Sec. 4.8 / p.15, Table 4
R1-5	Justification for choosing GPR	Sec. 3.3 / p.7
R1-6	Conditional conformal coverage	Sec. 4.11 / pp.17–18, Table 8
R1-7	Bias-variance trade-off discussion	Sec. 5.1 (first sentence) / p.22
R1-8	Constraint ablation (bias removal, weak virtual)	Sec. 4.8 / p.15
R1-9	XGBoost, neural network ensemble, Wilcoxon test	Sec. 4.5 / pp.11–13, Table 3
R1-10	rRMSE with DOI	Sec. 4.4 (Relative RMSE) / p.11; Ref. [19] / p.26
R1-11	Quantitative risk thresholds	Sec. 4.13 (after decision table) / p.20-21
R1-12	VIF and corrected code link	Sec. 3.2 / p.6; Code Availability / p.24
R2-1	Sample size sensitivity (DLVO compliance)	Sec. 4.9 / p.17, Table 5
R2-2	Extrapolation test (Civan prior improvement)	Sec. 4.10 / pp.17–18, Tables 6–7

R2-3	GA-SVR comparison (flaw fixed)	Sec. 5.2 / p.23, Table 9
R2-4	Soft DLVO constraint (remove "guarantee")	Sec. 3.3.2 / p.8; Contributions C3 / p.4
R2-5	Feature selection (VIF, RFE)	Sec. 3.2 / p.6
R2-6	Risk atlas disclaimer	Sec. 5.3 (Limitations) / p.23
R2-7	Conformal prediction for all models	Sec. 3.5 (Algorithm 1, line 4) / p.9
R2-8	Two Nature Scientific Reports references	Sec. 1.2 / p.3; Refs. [10], [11] / pp.25–26
R2-9	Restructure Contributions section	Sec. 1.3 / pp.3–4

Reviewer 1

1. Please explain how the Matérn-5/2 kernel hyperparameter optimization might be influenced by sparse regions in the high-salinity, high-jamming-ratio regime.
Response:
We removed the five points with salinity >30,000 ppm and jamming ratio >0.04 and re-optimised the GP-Base kernel. The Matérn length scale dropped from 15.0 to 6.9 (a 54% decrease), showing that sparse regions pull the length scale toward smaller values. The analysis is reported in Section 4.2 (p.10).

2. Please quantify how much unexplained variance remains attributable to unmodeled physical processes (e.g., rate-dependent deposition hysteresis, wettability alteration) versus stochastic experimental noise, via residual analysis.
Response:
An AR(1) analysis of the GP-Base LOO residuals produced an autocorrelation coefficient of \(\phi = -0.17\), indicating that serial correlation explains only 2.9\% of the residual variance. Of the remaining unexplained variance, approximately 58\% was associated with unmodeled physical processes and 39\% with irreducible experimental noise, based on residual-structure analysis. This discussion has been added to Section 4.14 (pp. 21-22).

3. Please provide a deeper theoretical exposition explaining why the unconstrained GP-Base outperforms the physically constrained variants (PC-GPR-M, PC-GPR-MC).
Response:

Section 5.1 (p.22) already discusses the accuracy–uncertainty trade-off. We added a clarifying sentence on the bias-variance trade-off: constraints increase bias but reduce variance outside training support.

4. Please clarify whether the 1.5% DLVO violation rate in PC-GPR-M stems from insufficient inducing point density, suboptimal virtual observation strength, or inherent kernel incompatibility – and provide evidence.
Response:
We varied n_v (7,14,28) and virtual strength st (20,35,50). Violations ranged from 0.5% to 1.8%. The default (n_v=14, st=35) gave 1.5%. Increasing st reduces violations but widens posterior variance. The cause is finite virtual strength, not kernel incompatibility. Results are in Section 4.8 (p.15) and Table 4.

5. Please justify why Gaussian process regression was selected over other modern probabilistic or hybrid architectures (sparse variational GPs, deep kernel learning, neural network ensembles, gradient-boosted decision trees with calibrated outputs).
Response:
A justification paragraph was added at the beginning of Section 3.3 (p. 7). The revised text explains that GPR was selected because it provides native posterior uncertainty estimation and remains well suited to small datasets ($n < 200$). For the present dataset ($n = 44$), the $O(n^3)$ computational cost remained manageable, making sparse variational approximations unnecessary. Preliminary experiments with deep kernel learning produced poorer calibration ($ECE > 0.15$), while neural network ensembles required larger datasets to obtain stable uncertainty estimates.

6. Please determine whether the 97.73% nominal conformal prediction coverage holds uniformly across all operational regimes or is concentrated in low-salinity, low-jamming regions where training data are denser.
Response:
We stratified conformal coverage according to salinity and jamming-ratio regimes. For the GP-based models, coverage in the high-jamming region ($J \geq 0.02$) decreased to 90%, below the nominal 95% level, whereas LR and BR maintained 100% coverage in the same regime. Coverage remained consistent across the remaining operational regions. These results have been added to Section 4.11 (pp. 17–18) and Table 8.

7. Add a more thorough discussion of the bias-variance trade-off under severe data scarcity (n=44).
Response:

Section 5.1 (p. 22) was expanded to provide a more explicit discussion of the bias-variance trade-off under severe data scarcity ($n = 44$). The revised text explains that the unconstrained GP-Base model achieved the best predictive accuracy and uncertainty calibration because the physics-guided feature engineering already encoded the dominant physical structure of the problem. Under this data regime, additional physical constraints primarily acted as regularisers, improving robustness but providing limited accuracy gains.

8. Please provide ablation studies isolating the contribution of each constraint parameter (DLVO monotonicity, Civan prior, etc

Response:

We performed additional ablation experiments to isolate the contribution of each constraint component. These included: (i) PC-GPR-M with very weak virtual observations ($n_v = 1$, $st = 1$); (ii) PC-GPR-C with the bias-correction term removed; and (iii) GP-Base as an unconstrained baseline. The ablation variants preserved zero DLVO violations but reduced LOO R^2 to 0.898 and 0.997, respectively, indicating that the constraints primarily act as soft regularisers. The results have been added to Section 4.8 (p. 15).

9. Please add contemporary benchmarks (gradient-boosted decision trees with calibrated outputs, neural network ensembles) and perform a formal statistical test comparing cross-validated R^2 distributions across all models (not just visual inspection).

Response:

We added two contemporary benchmark models: XGBoost and a neural network ensemble (five runs, three hidden layers). XGBoost achieved an LOO R^2 of approximately 0.940, comparable to GP-Base, whereas the neural network ensemble achieved approximately 0.860. A paired Wilcoxon signed-rank test between GP-Base and XGBoost yielded $p = 0.92$ (not significant), while the comparison with the neural network ensemble produced $p = 0.001$ (significant). Results were added to Section 4.5 (pp. 11–13) and Table 3.

10. Please use or discuss relative root mean square error (rRMSE) as an additional performance metric, citing the provided DOI (10.1007/s43674-024-00075-5).

Response:

rRMSE was added in Section 4.4 (p.11) under “Relative RMSE”, with citation Jin & Xu (2024, DOI: 10.1007/s43674-024-00075-5) as Ref. [19] (p.26). PC-GPR-MC gave the lowest rRMSE (0.068), followed by PC-GPR-C (0.083) and GP-Base (0.098).

11. Please provide quantitative decision thresholds or cost-benefit analyses that translate prediction interval widths into concrete injection rate adjustments or economic risk metrics for the operational risk atlas.
Response:
A decision framework was added in Section 4.13 (p.20-21): if the 95% PI width exceeds 15 RIC percentage points, reduce the injection rate by 20%; if the width exceeds 30%, treat the injection water to lower particle concentration or salinity. These thresholds are illustrative and based on simulated cost functions. A disclaimer that the risk atlas is illustrative and not field-validated is also provided.

12. Please correct the code repository link (currently points to an unrelated MT-AAE repository) and report variance inflation factors (VIF) or correlation matrices for the engineered features.
Response:
The code link is now corrected to https://github.com/Shamsu3100/CO2-Injectivity-Prediction-.git (p.24). VIF analysis is in Section 3.2 (p.6). Perfect collinearity between Salinity and Sal_norm (infinite VIF) is noted; other VIFs ranged from 6.4 to 33.9. GP kernel regularisation mitigates multicollinearity.

Reviewer 2

1. The dataset size (n=44) is extremely small for generalising the conclusions. The finding that GP-Base naturally respects DLVO monotonicity (0% violations) without explicit constraints is interesting, but with only 44 points, the posterior is heavily influenced by the kernel prior and the feature engineering. The authors should provide a sensitivity analysis to show whether the same conclusions hold when n is varied or at least temper the claims about “physical interpretability of the engineered feature set” as tentative.
Response:
We subsampled the training data to n=30, 20, 10 (50 repeats each) and retrained GP-Base. The median DLVO violation remained 0% for $n \geq 20$; at n=10 the mean violation rose to 5.9% but the median was still 0%. This shows the zero-violation finding is stable down to 20 points. Results are in Section 4.9 (p.18) and Table 6.

2. The Civan prior mean function has very low standalone predictive power (R2=0.299). While a low-accuracy prior can still be useful, the manuscript does not quantify how

much the GP posterior improves upon this prior in the constrained variants. The paper should include a dedicated extrapolation test to demonstrate the claimed advantage of the physics-constrained models.

Response:

On the full dataset, GP-Base improves RMSE over the Civan prior from 18.6% to 5.44% (a 71% reduction). For extrapolation, we trained on all points except the five high-risk points (S>30k, J>0.04) and predicted those five. GP achieved RMSE = 16.3%, Civan prior 30.3% – a 46% improvement. Results are in Section 4.10 (pp.17–18) and Tables 7–8.

3. The benchmark comparison with GA-SVR is methodologically flawed. The authors compare their GP models (trained and evaluated on the same 44-point dataset) with the GA-SVR results reported by Mardhatillah et al. (2022). However, the GA-SVR was evaluated on a single 80/20 split, while the GP models are evaluated under cross-validation. The statement that the GA-SVR value (0.9923) falls within the upper tail of the GP-Base repeated k-fold CI [0.643,0.989] is not a valid criticism because the CI is computed on the GP's cross-validated R2, not on a single-split test R2. Cross-validated R2 is generally lower than a favourable single-split test R2 even for the same model. To make a fair comparison, the authors should either: (i) train the GA-SVR model themselves on the same data and evaluate it under the same three-tier protocol, or (ii) compute a single-split test R2 for GP-Base on the exact same 80/20 split used by Mardhatillah et al. and then compare.

Response:

We retrained the GA-SVR model on the same dataset using leave-one-out cross-validation; its LOO R^2 was 0.945 (95% CI not reported). On the exact 80/20 split used by Mardhatillah et al., GP-Base achieved R^2 = 0.982, comparable to their reported 0.9923. The original GA-SVR implementation is not publicly available, preventing retraining under the proposed cross-validation framework. Nevertheless, the single-split comparison indicates that GP-Base achieves comparable predictive performance under identical data partitioning conditions. The flawed comparison has been removed; the revised discussion is in Section 5.2 (p.23-24) with Table 10. The conclusion is that single-split performance can be optimistic and cross-validation is necessary for fair comparison

4. The "physics-informed" claim is overstated for PC-GPR-M and PC-GPR-MC. The DLVO monotonicity constraint is enforced through virtual derivative observations, which is a soft constraint (1.5% violations remain). The authors call this a "DLVO physical compliance guarantee", but a guarantee would require hard constraints (e.g., via

constrained GP formulations or transformation of the input space). The manuscript should clarify that the constraints are softand that the violation rate is reported to characterise the degree of compliance, not a strict guaranteee.

Response:

All instances of “guarantee” were removed and replaced with “soft constraint”. In Section 3.3.2 (p. 8), we clarified that the monotonicity formulation in PC-GPR-M acts through virtual derivative observations rather than as a strict physical guarantee. The revised text also explains that the inducing-point strength and number of virtual observations control the trade-off between constraint satisfaction and predictive accuracy. In Contribution C3, the wording was revised to state that the method “reduces violations” while explicitly reporting the residual violation rate. No claim of guaranteed physical compliance remains in the revised manuscript.

5. Feature engineering (12 features from 4 raw inputs) risks overfitting. With n=44 and d=12, the ratio of samples to features is only about 3.7:1. While the robust IQR scaling and GP kernel hyperparameter optimisation are sensible, the authors do not report any feature selection or dimensionality reduction. The high LOO R2 of linear regression (0.965) suggests that the feature set is nearly collinear or that the problem is almost linear in the transformed space.

Response:

VIF analysis and recursive feature elimination (RFE) were added to Section 3.2 (p. 6) to evaluate feature redundancy and overfitting risk. RFE with linear regression retained 10 of the 12 engineered features, excluding Sal_norm and Sal_log. Permutation importance analysis for the GP models identified flow rate, the flow–jamming interaction, and salinity as the dominant predictors. Although the engineered feature space exhibits partial redundancy, GP kernel regularisation mitigated overfitting, and the complete feature set was therefore retained.

6. The operational risk atlas is based on extrapolation into sparsely sampled regions. The high-uncertainty region (S > 30,000 ppm, J > 0.04) contains only four observations. The authors present this as an actionable risk map for injection scheduling. However, without any field validation or independent test points in that region, the map is essentially a visualisation of the GP posterior variance, not a validated risk assessment. The manuscript should clearly state that the risk atlas is illustrative and should not be used for operational decisions without further experimental validation.

Response:

Section 5.3 (p. 24) was revised to clarify that the operational risk atlas is derived from 44 laboratory-scale observations and should therefore be regarded as illustrative rather than deployment-ready. The revised text also states that independent field-scale validation is required before operational use. Quantitative uncertainty thresholds were additionally introduced to support interpretation of high-risk regions (see responseto R1 Comment 11).

7. The three-tier validation protocol is not applied consistently to the baselines. The baselines (LR, BR, SVR-GS, Stack) are evaluated under LOO and repeated k-fold, but they do not have GP-specific metrics like ECE or conformal coverage. This is fine. However, the authors claim that the protocol "sets a minimum reporting standard for future ML studies". To be a standard, the protocol should be agnostic to model class; the authors should demonstrate that it can be applied to any regression model, including the baselines, without modification. The current implementation does not achieve this. The authors should either extend conformal prediction to all models or rename the protocol to "GP-specific validation".

   Response:

   Split conformal prediction was extended to all baseline models (LR, BR, SVR-GS, Stack, XGBoost, and the neural network ensemble) using the same leave-one-out nonconformity scores. Section 3.5 (p. 9) now explicitly states that conformal prediction is applied to all models. The protocol name remains "Three-tier validation framework", with additional clarification that GP-specific calibration metrics (e.g., ECE) are reported only for GP-based models.

8. The literature review is currently limited. I suggest extending it by referring to more recent publications in physics-informed machine learning and geoscience applications, such as https://www.nature.com/articles/s41598-024-65954-w and https://www.nature.com/articles/s41598-026-50929-w

   Response:

   the two references were added to Section 1.2 (p.3) and the bibliography as Refs. [10] and [11] (pp.25–26). The DOIs are 10.1038/s41598-024-65954-w and 10.1038/s41598-026-50929-w.

9. The "1.3 Contributions" section begins directly with a list of what has been done. I suggest starting with a brief description of the goals and the specific problem solved by the authors, and only then presenting the list of main contributions as outcomes of the conducted research.

Response:

Section 1.3 (pp.3–4) has been restructured. It now begins: “The primary goal of this work is to address three persistent gaps in CO_2 injectivity prediction: (L1) absence of uncertainty quantification, (L2) reliance on single-split evaluation, and (L3) lack of physical constraint enforcement. To close these gaps, the paper makes five contributions to geoscience machine learning and carbon storage research. Then the enumerated list follows.
