# Supplementary material for: Physics-informed gaussian process regression for reproducible and uncertainty-aware CO2 injectivity prediction
Source: PLoS One. 2026 Jul 1;21(7):e0352178. doi: 10.1371/journal.pone.0352178 (PMC13322525; doi:10.1371/journal.pone.0352178)
Supplement: Peer review history

# Peer review: Decision Letter 0

**DOI:** 10.1371/journal.pone.0352178.r001

**Authors:** Baohua Guo

19 May 2026

PONE-D-26-17436

Physics-Informed Gaussian Process Regression for Reproducible and Uncertainty-Aware CO2 Injectivity Prediction

PLOS One

Dear Dr. Adamu,

Thank you for submitting your manuscript to PLOS ONE. After careful consideration, we feel that it has merit but does not fully meet PLOS ONE’s publication criteria as it currently stands. Therefore, we invite you to submit a revised version of the manuscript that addresses the points raised during the review process.

Please submit your revised manuscript by Jul 03 2026 11:59PM. If you will need more time than this to complete your revisions, please reply to this message or contact the journal office at plosone@plos.org. When you're ready to submit your revision, log on to https://www.editorialmanager.com/pone/ and select the 'Submissions Needing Revision' folder to locate your manuscript file.

Please include the following items when submitting your revised manuscript:

- A letter that responds to each point raised by the academic editor and reviewer(s). You should upload this letter as a separate file labeled 'Response to Reviewers'.
- A marked-up copy of your manuscript that highlights changes made to the original version. You should upload this as a separate file labeled 'Revised Manuscript with Track Changes'.
- An unmarked version of your revised paper without tracked changes. You should upload this as a separate file labeled 'Manuscript'.

If you would like to make changes to your financial disclosure, please include your updated statement in your cover letter. Guidelines for resubmitting your figure files are available below the reviewer comments at the end of this letter.

If applicable, we recommend that you deposit your laboratory protocols in protocols.io to enhance the reproducibility of your results. Protocols.io assigns your protocol its own identifier (DOI) so that it can be cited independently in the future. For instructions see: https://journals.plos.org/plosone/s/submission-guidelines#loc-laboratory-protocols. Additionally, PLOS ONE offers an option for publishing peer-reviewed Lab Protocol articles, which describe protocols hosted on protocols.io. Read more information on sharing protocols at https://plos.org/protocols?utm_medium=editorial-email&utm_source=authorletters&utm_campaign=protocols.

As the corresponding author, your ORCID iD is verified in the submission system and will appear in the published article. PLOS supports the use of ORCID, and we encourage all coauthors to register for an ORCID iD and use it as well. Please encourage your coauthors to verify their ORCID iD within the submission system before final acceptance, as unverified ORCID iDs will not appear in the published article. *Only*  the individual author can complete the verification step; PLOS staff *cannot*  verify ORCID iDs on behalf of authors.

We look forward to receiving your revised manuscript.

Kind regards,

Baohua Guo

Academic Editor

PLOS One

Journal Requirements:

When submitting your revision, we need you to address these additional requirements.

1.Please ensure that your manuscript meets PLOS ONE's style requirements, including those for file naming. The PLOS ONE style templates can be found at

https://journals.plos.org/plosone/s/file?id=wjVg/PLOSOne_formatting_sample_main_body.pdf and

https://journals.plos.org/plosone/s/file?id=ba62/PLOSOne_formatting_sample_title_authors_affiliations.pdf

2. Please note that funding information should not appear in any section or other areas of your manuscript. We will only publish funding information present in the Funding Statement section of the online submission form. Please remove any funding-related text from the manuscript.

3. We note that the grant information you provided in the ‘Funding Information’ and ‘Financial Disclosure’ sections do not match.

When you resubmit, please ensure that you provide the correct grant numbers for the awards you received for your study in the ‘Funding Information’ section

4. Please provide a complete Data Availability Statement in the submission form, ensuring you include all necessary access information or a reason for why you are unable to make your data freely accessible. If your research concerns only data provided within your submission, please write "All data are in the manuscript and/or supporting information files" as your Data Availability Statement.

5. Please amend your authorship list in your manuscript file to include author Dr. Majdy Mohamed Eltayeb Eltahir.

6. If the reviewer comments include a recommendation to cite specific previously published works, please review and evaluate these publications to determine whether they are relevant and should be cited. There is no requirement to cite these works unless the editor has indicated otherwise.

[Note: HTML markup is below. Please do not edit.]

Reviewers' comments:

Reviewer's Responses to Questions

**Comments to the Author**

1. Is the manuscript technically sound, and do the data support the conclusions?

The manuscript must describe a technically sound piece of scientific research with data that supports the conclusions. Experiments must have been conducted rigorously, with appropriate controls, replication, and sample sizes. The conclusions must be drawn appropriately based on the data presented.

Reviewer #1: Partly

Reviewer #2: Yes

**********

2. Has the statistical analysis been performed appropriately and rigorously?

Reviewer #1: Yes

Reviewer #2: Yes

**********

3. Have the authors made all data underlying the findings in their manuscript fully available?

The PLOS Data policy requires authors to make all data underlying the findings described in their manuscript fully available without restriction, with rare exception (please refer to the Data Availability Statement in the manuscript PDF file). The data should be provided as part of the manuscript or its supporting information, or deposited to a public repository. For example, in addition to summary statistics, the data points behind means, medians and variance measures should be available. If there are restrictions on publicly sharing data—e.g. participant privacy or use of data from a third party—those must be specified.

Reviewer #1: Yes

Reviewer #2: Yes

**********

4. Is the manuscript presented in an intelligible fashion and written in standard English?

PLOS ONE does not copyedit accepted manuscripts, so the language in submitted articles must be clear, correct, and unambiguous. Any typographical or grammatical errors should be corrected at revision, so please note any specific errors here.

Reviewer #1: Yes

Reviewer #2: Yes

**********

5. Review Comments to the Author

Please use the space provided to explain your answers to the questions above. You may also include additional comments for the author, including concerns about dual publication, research ethics, or publication ethics. (Please upload your review as an attachment if it exceeds 20,000 characters)

Reviewer #1: The manuscript presents a timely and methodologically rigorous investigation into physics-informed Gaussian process regression for predicting CO2 injectivity decline, addressing critical gaps in uncertainty quantification, reproducible evaluation, and physical constraint enforcement within geological carbon storage applications. The integration of DLVO colloidal monotonicity and the Civan–Kozeny–Carman permeability impairment model into a Gaussian process framework represents a thoughtful advancement over purely data-driven approaches, and the implementation of a three-tier validation protocol combining leave-one-out cross-validation, repeated k-fold cross-validation, and non-parametric bootstrap confidence intervals establishes a commendable standard for statistical robustness in small-sample geoscience studies. The authors’ emphasis on uncertainty-aware modeling, particularly through the combination of GP posterior calibration using Expected Calibration Error and split-conformal prediction intervals, is highly valuable for operational decision-making in carbon capture and storage, where risk quantification is as crucial as point prediction accuracy. The operational risk atlas translating predictive uncertainty into actionable injection scheduling guidance effectively bridges the gap between laboratory-scale modeling and field deployment, and the transparent acknowledgment of the accuracy–uncertainty trade-off inherent in physics-informed modeling at limited data densities demonstrates scholarly maturity. However, despite these substantial strengths, the manuscript requires major revision to address several methodological, contextual, and evaluative shortcomings before it can meet the publication standards of PLOS ONE. The most pressing concern revolves around the extremely limited sample size of n=44 laboratory measurements, which inherently restricts the statistical power and generalizability of all reported models. While the authors appropriately employ rigorous resampling techniques to mitigate overfitting, the fundamental constraint of learning complex nonlinear interactions across four primary input variables and eight engineered features from fewer than fifty observations warrants more cautious interpretation of the reported R2 values and confidence intervals. The manuscript would benefit significantly from a more thorough discussion of the bias-variance trade-off under such severe data scarcity, particularly regarding how the Matérn-5/2 kernel hyperparameter optimization might be influenced by sparse regions in the high-salinity, high-jamming-ratio regime. Furthermore, the reliance on a single curated laboratory dataset from prior literature introduces potential batch effects and experimental design limitations that are not fully accounted for in the uncertainty propagation framework; a sensitivity analysis demonstrating how model performance degrades under systematic perturbations or subsampling strategies would strengthen the claim of robustness. Regarding the physics-informed components, the observation that the unconstrained GP-Base model achieves superior leave-one-out accuracy and calibration compared to the constrained variants is an important empirical finding, yet the underlying mechanism driving this result requires deeper theoretical exposition. The authors attribute the slight performance degradation in PC-GPR-M and PC-GPR-MC to constraint tension and reduced posterior flexibility, but a more rigorous analysis of how virtual derivative observations and prior mean functions interact with the GP marginal likelihood optimization process would be highly instructive. Specifically, the manuscript should clarify whether the 1.5% DLVO violation rate in PC-GPR-M stems from insufficient inducing point density, suboptimal virtual observation strength, or inherent kernel incompatibility, and provide ablation studies isolating the contribution of each constraint parameter. Additionally, the claim that domain-guided feature engineering implicitly encodes DLVO monotonicity, while empirically supported by the zero violation rate in GP-Base, should be contextualized within the broader literature on implicit versus explicit physics integration, particularly regarding how engineered interaction terms like Bidx and S·Q approximate underlying pore-scale electrostatic and hydrodynamic mechanisms without guaranteeing thermodynamic consistency. Enhance connections to recent literature that demonstrates the great potential and use of several machine learning models, such as the neural network (doi: 10.1142/S2737599425500367), Gaussian process regression (doi: 10.1080/17509653.2025.2453902; 10.1007/s00521-024-10726-w), and ensemble/composite method (doi: 10.1002/ajae.12041), for modelling complicated (nonlinear) patterns across a broad variety of study subjects in order to further motivate the exploration of machine learning models in your present work. Building upon this contextual expansion, the manuscript’s comparative analysis should explicitly address why Gaussian process regression was selected over other modern probabilistic or hybrid architectures that have demonstrated comparable or superior uncertainty quantification capabilities in similar geoscientific domains. While the O(n3) computational cost is manageable for n=44, the authors should discuss scalability considerations and potential alternatives such as sparse variational Gaussian processes or deep kernel learning, which could better accommodate future experimental expansions. The baseline model selection, comprising Linear Regression, Bayesian Ridge, grid-search SVR, and a stacking ensemble, is adequate but somewhat dated; incorporating more contemporary benchmarks such as gradient-boosted decision trees with calibrated outputs or neural network ensembles would provide a more comprehensive performance landscape. Moreover, the treatment of the published GA-SVR benchmark as a single-split upper-tail outcome is statistically sound, yet the manuscript would be strengthened by a formal statistical test comparing the distribution of cross-validated R2 scores across all models rather than relying solely on visual inspection of confidence interval overlap. Use or at least discuss other performance measures, such as the relative root mean square error, that facilitate performance comparisons across models or targets (see doi: 10.1007/s43674-024-00075-5). In conjunction with expanding the evaluation metrics, the authors should provide a more nuanced interpretation of the Average Absolute Percentage Error values, which appear disproportionately high relative to the reported R2 scores, suggesting potential scale-dependency issues or outlier sensitivity that warrants explicit diagnostic testing. The conformal prediction framework is correctly implemented and provides valuable finite-sample coverage guarantees, but the manuscript lacks a discussion on conditional versus marginal coverage, which is particularly relevant given the heterogeneous uncertainty structure across the salinity-jamming ratio space. Operators deploying this model in field conditions would benefit from understanding whether the 97.73% nominal coverage holds uniformly across different operational regimes or is concentrated in low-salinity, low-jamming regions where training data are denser. Furthermore, the operational risk atlas, while conceptually excellent, should be accompanied by quantitative decision thresholds or cost-benefit analyses that translate prediction interval widths into concrete injection rate adjustments or economic risk metrics, thereby enhancing its practical utility for reservoir engineers. The technical presentation of the methodology is generally clear, but several aspects of the implementation require greater transparency to ensure full reproducibility. The manuscript should specify the exact random seeds, optimization algorithms, and convergence criteria used for hyperparameter tuning, particularly regarding the eight random restarts for log-marginal likelihood maximization and the multi-start L-BFGS-B optimization for the Civan prior parameters. The feature engineering process, while physically motivated, introduces derived variables that may induce multicollinearity, and the authors should report variance inflation factors or correlation matrices to demonstrate that robust scaling adequately mitigates numerical instability in the GP kernel computations. Additionally, the code availability statement references an MT-AAE repository, which appears unrelated to the Gaussian process framework described in the manuscript; this discrepancy must be corrected to point to the actual CO2 injectivity prediction repository, and all preprocessing pipelines, constraint implementations, and conformal calibration routines should be fully documented. The limitations section appropriately acknowledges the sparse experimental grid, the modest standalone explanatory power of the Civan prior, and the absence of field-scale validation, but these constraints should be more rigorously integrated into the main discussion rather than relegated to a brief subsection. The manuscript would significantly benefit from a prospective experimental design recommendation specifying how additional laboratory measurements should be strategically allocated across the input space to maximize information gain, particularly in the high-uncertainty region identified at salinity > 30,000 ppm and jamming ratio > 0.04. Furthermore, the coupling of rate-dependent deposition hysteresis and wettability alteration into the mechanistic prior is mentioned as future work, but the current formulation’s inability to capture these dynamics should be explicitly quantified through residual analysis, demonstrating how much unexplained variance remains attributable to unmodeled physical processes versus stochastic experimental noise. In summary, while the manuscript delivers a novel, uncertainty-aware, and physically constrained modeling framework that advances the state of the art in CO2 injectivity prediction, it requires substantial revisions to strengthen its theoretical grounding, expand its comparative and evaluative scope, clarify its implementation details, and enhance its practical relevance for subsurface engineering applications. Addressing these concerns through methodological refinements, broader literature contextualization, expanded performance diagnostics, and corrected reproducibility artifacts will significantly elevate the manuscript’s scientific rigor and operational impact. I recommend major revision to allow the authors the opportunity to comprehensively address these points, after which the work will be well-positioned for publication as a robust and reproducible contribution to physics-informed machine learning in geological carbon storage.

Reviewer #2: The manuscript addresses an important practical problem of uncertainty‑aware prediction of CO2 injectivity and makes a valuable contribution by introducing conformal prediction and uncertainty quantification to this domain. The three‑tier validation is a step forward from single‑split reporting. However, the paper overstates the benefits of physics constraints given the small dataset and lack of extrapolation validation, and the comparison with the GA‑SVR benchmark is not methodologically sound. The authors should justify or make less strong claims about “guarantees” and “operational risk maps”, clarify the limitations of the small dataset, and add extrapolation experiments to support the claimed advantage of constrained models. With these revisions, the work would be a valuable resource for the CCS and geoscience machine learning communities.

1) The dataset size (n=44) is extremely small for generalising the conclusions. The finding that GP‑Base naturally respects DLVO monotonicity (0% violations) without explicit constraints is interesting, but with only 44 points, the posterior is heavily influenced by the kernel prior and the feature engineering. The authors should provide a sensitivity analysis to show whether the same conclusions hold when n is varied, or at least temper the claims about “physical interpretability of the engineered feature set” as tentative.

2) The Civan prior mean function has very low standalone predictive power (R2=0.299). While a low‑accuracy prior can still be useful, the manuscript does not quantify how much the GP posterior improves upon this prior in the constrained variants. The paper should include a dedicated extrapolation test to demonstrate the claimed advantage of the physics‑constrained models.

3) The benchmark comparison with GA‑SVR is methodologically flawed. The authors compare their GP models (trained and evaluated on the same 44‑point dataset) with the GA‑SVR results reported by Mardhatillah et al. (2022). However, the GA‑SVR was evaluated on a single 80/20 split, while the GP models are evaluated under cross‑validation. The statement that the GA‑SVR value (0.9923) falls within the upper tail of the GP‑Base repeated k‑fold CI [0.643,0.989] is not a valid criticism because the CI is computed on the GP’s cross‑validated R2, not on a single‑split test R2. Cross‑validated R2 is generally lower than a favourable single‑split test R2 even for the same model. To make a fair comparison, the authors should either: (i) train the GA‑SVR model themselves on the same data and evaluate it under the same three‑tier protocol, or (ii) compute a single‑split test R2 for GP‑Base on the exact same 80/20 split used by Mardhatillah et al. and then compare.

4) The “physics‑informed” claim is overstated for PC‑GPR‑M and PC‑GPR‑MC. The DLVO monotonicity constraint is enforced through virtual derivative observations, which is a soft constraint (1.5% violations remain). The authors call this a “DLVO physical compliance guarantee”, but a guarantee would require hard constraints (e.g., via constrained GP formulations or transformation of the input space). The manuscript should clarify that the constraints are softand that the violation rate is reported to characterise the degree of compliance, not a strict guarantee.

5) Feature engineering (12 features from 4 raw inputs) risks overfitting. With n=44 and d=12, the ratio of samples to features is only about 3.7:1. While the robust IQR scaling and GP kernel hyperparameter optimisation are sensible, the authors do not report any feature selection or dimensionality reduction. The high LOO R2 of linear regression (0.965) suggests that the feature set is nearly collinear or that the problem is almost linear in the transformed space.

6) The operational risk atlas is based on extrapolation into sparsely sampled regions. The high‑uncertainty region (S > 30,000 ppm, J > 0.04) contains only four observations. The authors present this as an actionable risk map for injection scheduling. However, without any field validation or independent test points in that region, the map is essentially a visualisation of the GP posterior variance, not a validated risk assessment. The manuscript should clearly state that the risk atlas is illustrative and should not be used for operational decisions without further experimental validation.

7) The three‑tier validation protocol is not applied consistently to the baselines. The baselines (LR, BR, SVR‑GS, Stack) are evaluated under LOO and repeated k‑fold, but they do not have GP‑specific metrics like ECE or conformal coverage. This is fine. However, the authors claim that the protocol “sets a minimum reporting standard for future ML studies”. To be a standard, the protocol should be agnostic to model class; the authors should demonstrate that it can be applied to any regression model, including the baselines, without modification. The current implementation does not achieve this. The authors should either extend conformal prediction to all models or rename the protocol to “GP‑specific validation”.

Minor issues:

8) The literature review is currently limited. I suggest extending it by referring to more recent publications in physics‑informed machine learning and geoscience applications, such as https://www.nature.com/articles/s41598-024-65954-w and https://www.nature.com/articles/s41598-026-50929-w

9) The “1.3 Contributions” section begins directly with a list of what has been done. I suggest starting with a brief description of the goals and the specific problem solved by the authors, and only then presenting the list of main contributions as outcomes of the conducted research.

**********

6. PLOS authors have the option to publish the peer review history of their article (what does this mean?). If published, this will include your full peer review and any attached files.

If you choose “no”, your identity will remain anonymous but your review may still be made public.

**Do you want your identity to be public for this peer review?**  For information about this choice, including consent withdrawal, please see our Privacy Policy.

Reviewer #1: No

Reviewer #2: No

**********

[NOTE: If reviewer comments were submitted as an attachment file, they will be attached to this email and accessible via the submission site. Please log into your account, locate the manuscript record, and check for the action link "View Attachments". If this link does not appear, there are no attachment files.]

To ensure your figures meet our technical requirements, please review our figure guidelines: https://journals.plos.org/plosone/s/figures

You may also use PLOS’s free figure tool, NAAS, to help you prepare publication quality figures: https://journals.plos.org/plosone/s/figures#loc-tools-for-figure-preparation.

NAAS will assess whether your figures meet our technical requirements by comparing each figure against our figure specifications.

# Author comment: Author response to Decision Letter 1

**DOI:** 10.1371/journal.pone.0352178.r002

25 May 2026

Editor

1. Please ensure that your manuscript meets PLOS ONE's style requirements, including those for file naming. The PLOS ONE style templates can be found at https://journals.plos.org/plosone/s/file?id=wjVg/PLOSOne_formatting_sample_main_body.pdf and https://journals.plos.org/plosone/s/file?id=ba62/PLOSOne_formatting_sample_title_authors_affiliations.pdf

Response: We have carefully reviewed both templates and formatted our manuscript accordingly. The main body now follows the required structure (sections, headings, references, figure citations, and file naming). The title, author list, and affiliations page also conform to the PLOS ONE specifications.

2. Please note that funding information should not appear in any section or other areas of your manuscript. We will only publish funding information present in the Funding Statement section of the online submission form. Please remove any funding-related text from the manuscript.

Response: All funding related text has been removed from the manuscript file (PDF and source). No funding information appears in the abstract, main text, acknowledgements, or any other section. The funding statement is provided in the Funding Statement section of the online submission form.

3. We note that the grant information you provided in the ‘Funding Information’ and ‘Financial Disclosure’ sections do not match. When you resubmit, please ensure that you provide the correct grant numbers for the awards you received for your study in the ‘Funding Information’ section.

Response: We have reconciled the grant information. The correct grant numbers are: [insert your correct grant numbers here (Grant No. RGP.2/209/46), YUTP-PRG 015PBC-028) . These same numbers now appear in both the ‘Funding Information’ and ‘Financial Disclosure’ sections of the online submission form.

4. Please provide a complete Data Availability Statement in the submission form, ensuring you include all necessary access information or a reason for why you are unable to make your data freely accessible. If your research concerns only data provided within your submission, please write "All data are in the manuscript and/or supporting information files" as your Data Availability Statement.

Response: Our complete Data Availability Statement is:

“All data are in the manuscript and/or supporting information files.”

The full dataset (44 experimental points) is listed in the appendix of the manuscript. The code used for analysis and model training is publicly available at the GitHub repository provided in the Code Availability section.

5. Please amend your authorship list in your manuscript file to include author Dr. Majdy Mohamed Eltayeb Eltahir.

Response: Dr. Majdy Mohamed Eltayeb Eltahir has been added to the authorship list in the manuscript file. The full author order has been updated accordingly.

6. If the reviewer comments include a recommendation to cite specific previously published works, please review and evaluate these publications to determine whether they are relevant and should be cited. There is no requirement to cite these works unless the editor has indicated otherwise.

Response: We have reviewed the publications recommended by the reviewers. Those that are directly relevant to our methodology or provide essential context have been cited.

Attachment Submitted filename: 2Reviwer_response_22May2026.pdf

# Peer review: Decision Letter 1

**DOI:** 10.1371/journal.pone.0352178.r003

**Authors:** Baohua Guo, Baohua Guo

8 Jun 2026

Physics-Informed Gaussian Process Regression for Reproducible and Uncertainty-Aware CO2 Injectivity Prediction

PONE-D-26-17436R1

Dear Dr. Adamu,

We’re pleased to inform you that your manuscript has been judged scientifically suitable for publication and will be formally accepted for publication once it meets all outstanding technical requirements.

Within one week, you’ll receive an e-mail detailing the required amendments. When these have been addressed, you’ll receive a formal acceptance letter and your manuscript will be scheduled for publication.

An invoice will be generated when your article is formally accepted. Please note, if your institution has a publishing partnership with PLOS and your article meets the relevant criteria, all or part of your publication costs will be covered. Please make sure your user information is up-to-date by logging into Editorial Manager at Editorial Manager® and clicking the ‘Update My Information' link at the top of the page. For questions related to billing, please contact billing support.

If your institution or institutions have a press office, please notify them about your upcoming paper to help maximize its impact. If they’ll be preparing press materials, please inform our press team as soon as possible -- no later than 48 hours after receiving the formal acceptance. Your manuscript will remain under strict press embargo until 2 pm Eastern Time on the date of publication. For more information, please contact onepress@plos.org.

Kind regards,

Baohua Guo

Academic Editor

PLOS One

Additional Editor Comments (optional):

Reviewers' comments:

Reviewer's Responses to Questions

**Comments to the Author**

1. If the authors have adequately addressed your comments raised in a previous round of review and you feel that this manuscript is now acceptable for publication, you may indicate that here to bypass the “Comments to the Author” section, enter your conflict of interest statement in the “Confidential to Editor” section, and submit your "Accept" recommendation.

Reviewer #1: (No Response)

**********

2. Is the manuscript technically sound, and do the data support the conclusions?

The manuscript must describe a technically sound piece of scientific research with data that supports the conclusions. Experiments must have been conducted rigorously, with appropriate controls, replication, and sample sizes. The conclusions must be drawn appropriately based on the data presented.

Reviewer #1: (No Response)

**********

3. Has the statistical analysis been performed appropriately and rigorously?

Reviewer #1: (No Response)

**********

4. Have the authors made all data underlying the findings in their manuscript fully available?

The PLOS Data policy requires authors to make all data underlying the findings described in their manuscript fully available without restriction, with rare exception (please refer to the Data Availability Statement in the manuscript PDF file). The data should be provided as part of the manuscript or its supporting information, or deposited to a public repository. For example, in addition to summary statistics, the data points behind means, medians and variance measures should be available. If there are restrictions on publicly sharing data—e.g. participant privacy or use of data from a third party—those must be specified.

Reviewer #1: (No Response)

**********

5. Is the manuscript presented in an intelligible fashion and written in standard English?

PLOS ONE does not copyedit accepted manuscripts, so the language in submitted articles must be clear, correct, and unambiguous. Any typographical or grammatical errors should be corrected at revision, so please note any specific errors here.

Reviewer #1: (No Response)

**********

6. Review Comments to the Author

Please use the space provided to explain your answers to the questions above. You may also include additional comments for the author, including concerns about dual publication, research ethics, or publication ethics. (Please upload your review as an attachment if it exceeds 20,000 characters)

Reviewer #1: The authors have adequately addressed all of my previous comments. I recommend that the manuscript be accepted for publication in its current form.

**********

7. PLOS authors have the option to publish the peer review history of their article (what does this mean?). If published, this will include your full peer review and any attached files.

If you choose “no”, your identity will remain anonymous but your review may still be made public.

**Do you want your identity to be public for this peer review?**  For information about this choice, including consent withdrawal, please see our Privacy Policy.

Reviewer #1: No

**********

# Editor report: Acceptance letter

**DOI:** 10.1371/journal.pone.0352178.r004

**Authors:** Baohua Guo, Baohua Guo

PONE-D-26-17436R1

PLOS One

Dear Dr. Adamu,

I'm pleased to inform you that your manuscript has been deemed suitable for publication in PLOS One. Congratulations! Your manuscript is now being handed over to our production team.

At this stage, our production department will prepare your paper for publication. This includes ensuring the following:

* All references, tables, and figures are properly cited

* All relevant supporting information is included in the manuscript submission,

* There are no issues that prevent the paper from being properly typeset

You will receive further instructions from the production team, including instructions on how to review your proof when it is ready. Please keep in mind that we are working through a large volume of accepted articles, so please give us a few days to review your paper and let you know the next and final steps.

Lastly, if your institution or institutions have a press office, please let them know about your upcoming paper now to help maximize its impact. If they'll be preparing press materials, please inform our press team within the next 48 hours. Your manuscript will remain under strict press embargo until 2 pm Eastern Time on the date of publication. For more information, please contact onepress@plos.org.

You will receive an invoice from PLOS for your publication fee after your manuscript has reached the completed accept phase. If you receive an email requesting payment before acceptance or for any other service, this may be a phishing scheme. Learn how to identify phishing emails and protect your accounts at https://explore.plos.org/phishing.

If we can help with anything else, please email us at customercare@plos.org.

Thank you for submitting your work to PLOS ONE and supporting open access.

Kind regards,

PLOS ONE Editorial Office Staff

on behalf of

Dr. Baohua Guo

Academic Editor

PLOS One
